# Synthesis, biological activities, DFT, and molecular docking of 1,3,4-thiadiazolo[2,3-c]-1,2,4-triazine-Palladium (II) complex

**DOI:** 10.1038/s41598-026-53122-1

**Published:** 2026-06-04

**Authors:** Safaa S. Hassan, Kamal M. Dawood, Amrajaa S. Abubakr, Naglaa S. Mahmoud, Nabila A. Kheder

**Affiliations:** 1 Department of Chemistry, Faculty of Science, Cairo University, Giza, 12613 Egypt; 2 Department of Chemistry, Faculty of Science, University of Ajdabiya, Ajdabiya, Libya; 3 Department of Industrial Pharmacy, College of Pharmaceutical Sciences and Drug Manufacturing, Misr University for Science and Technology, P.O. 12566, Giza, Egypt

**Keywords:** [1,3,4]thiadiazole-[1,2,4]triazine, Pd(II) complex, Regioselective synthesis, Antimicrobial activity, Anticancer activity, Computational studies, Biochemistry, Biotechnology, Chemical biology, Chemistry, Drug discovery, Microbiology

## Abstract

A developed synthesis of 4-amino-6-methyl-3-thioxo-3,4-dihydro-1,2,4-triazin-5(2 H)-one (**2**) was presented. Its reaction with PhNCS in ethanol afforded 3-methyl-7-(phenylamino)-4 H-[1,3,4]thiadiazolo[2,3-c][1,2,4]triazin-4-one (L) via an unexpected mechanism. A combination of different characterization techniques (CHN, IR, NMR, UV-Vis TGA, XRD and MS) was utilized for comprehensive structure elucidation. [Pd(L)Cl_2_].4DMF complex was obtained by preparing the Pd(II) complex at a molar ratio of 1:1 with respect to Pd(II): L. DFT simulated several proposed intermediates for (L) production. Furthermore, DFT corroborated the square planar geometric configuration of the synthesized Pd(II) complex. In vitro, the antimicrobial activity of the synthesized compound on *Escherichia coli*, *Klebsiella pneumoniae*, *Staphylococcus aureus*, *Streptococcus mutans*,* Candida albicans*, *and Aspergillus Nigar* using the inhibition zone technique was evaluated. Subsequent determination of the minimum inhibitory concentration (MIC) of the Pd complex against *Klebsiella* confirmed its potent antibacterial activity, in agreement with the pronounced inhibition zone observed in the diffusion assay. The results indicate that the palladium (II) complex is more potent than the ligand against the tested microorganisms, especially against *K. pneumoniae*, where it demonstrated promising activity that, upon further research, could be used to overcome gentamicin resistance. Both compounds exhibited similar cytotoxicity against the proliferation of the human liver cancer cell line. The interactions at the docking active sites were assessed using the chosen proteins, EGFR tyrosine kinase, and the protein crystal structure of GlcN-O-P synthase. The anti-inflammatory effect results indicated that both compounds have a moderate effect on bovine serum albumin (BSA).

**Supplementary Information:**

The online version contains supplementary material available at 10.1038/s41598-026-53122-1.

## Introduction

**Fig. 1:**
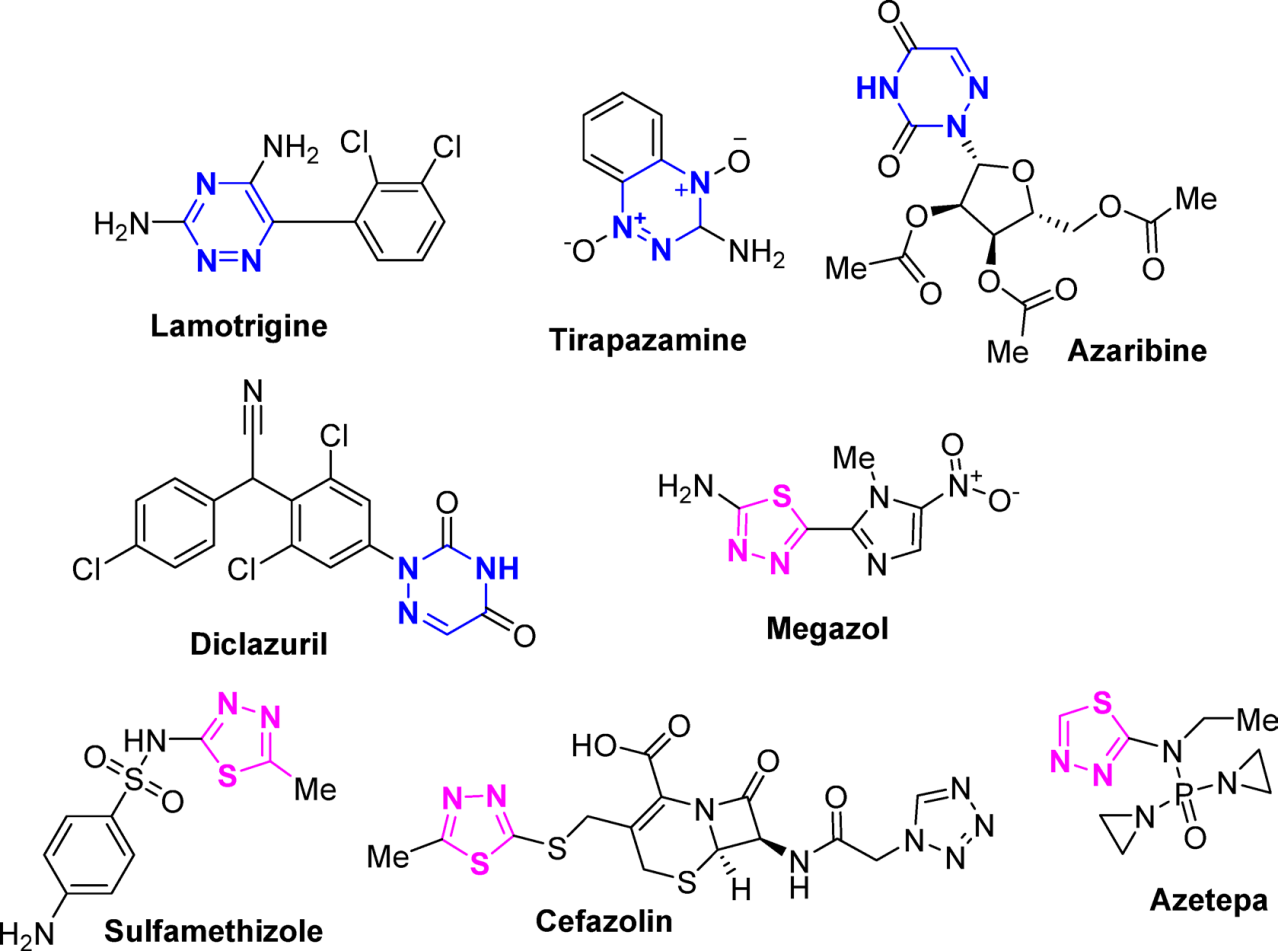
Structures of some drugs containing 1,2,4-triazine or 1,3,4-thiadiazole cores.

Triazine compounds contain a 6-membered ring with three nitrogen atoms that may be symmetric or asymmetric based on the arrangement of nitrogen atoms. Out of the three classes of triazines, a plethora of 1,2,4-triazines or *as*-triazines have been delineated to show significant biological, pharmacological, and medicinal properties such as antifungal^1^, antiinflammatory^2^, antihypertensive^3^, antimalarial^4,5^, antibacterial^6,7^, anti-HIV^8,9^, and anticancer^9^. Also, the 1,2,4-triazine nucleus is present in many marketed drugs^10^ (Fig. 1).

1,3,4-Thiadiazole derivatives exhibit tremendous biological activities^11–13^, including anticancer, antimicrobial, anti-tuberculosis, antiinflammatory, antiviral, antileishmanial, and antiepileptic. Additionally, many medications contain a 1,2,4-thiadiazole moiety, such as the antiprotozoal *Megazol*, the antimicrobial *Sulphamethizole*, the anticancer *Azetepa*, and the antibiotic *Cefazolin* (Fig. 1).

Encouraged by these results, and in continuation of our previous studies for hybridizing bioactive heterocyclic rings^14–19^, we report herein the reaction of 4-amino-6-methyl-3-thioxo-3,4-dihydro-1,2,4-triazin-5(2 H)-one (**1**), obtained from the stirring of thiocarbohydrazide with sodium pyruvate at room temperature, with PhNCS in ethanol. Interestingly, the latter reaction yielded thiadiazolo-triazine hybrid **5**. The regioselectivity of its preparation and reactivity as a ligand and its new Pd(II)-complex of **5** were studied in addition to their activities as possible antibacterial, antifungal, and antitumor agents.

It is well known that the coordination of many metal ions and ligands enhances the biological activities through chelation with platinum-group metals^20–24^. Palladium is one of these metals whose complexes exhibit a noticeable cytotoxic activity similar to that of standard platinum-based drugs such as *cisplatin*, *carboplatin*, and *oxaliplatin*, with fewer side effects than other heavy-metal anticancer compounds^25–27^. Also, the epidermal growth factor receptor (EGFR) is a transmembrane glycoprotein that belongs to the family of tyrosine kinase receptors (TKRs). Mice manganese-lacking EGFR show delayed regeneration after partial hepatectomy (PH), demonstrating that EGFR is a critical regulator of hepatocyte proliferation during liver regeneration^28^. Hence, EGFR has long been an attractive anticancer drug target. Therefore, we can predict the mode of action of the synthesized compounds as EGFR inhibitors using molecular docking. Considering all of the above, our work aimed to examine the effects of triazine ligand and its novel Pd(II) complex as possible antibacterial and liver anticancer agents, with further rationalization of the biological results by performing molecular docking on the synthesized compounds to predict the best mode by which a compound will fit into a binding site of a macromolecular target.

## Experimental

### Chemistry section

#### Instruments

Supplementary file (SI file) contains details of all analytical and spectroscopic instruments employed in the characterization of the synthesized compounds.

#### Materials and purification

All solvents and reagents were purchased from trusted commercial stores, including Aldrich (purity > 99%, St. Louis, MO, USA), and used without further purification. The remaining chemicals utilized in the work were of analytical reagent grade.

#### Synthetic procedure

##### Synthesis of 4-amino-6-methyl-3-thioxo-3,4-dihydro-1,2,4-triazin-5(2 H)-one (1)

To a solution of thiocarbohydrazide (1.06 g, 10 mmol) in H_2_O (10 mL) was added sodium pyruvate (1.10 g, 10 mmol), then dilute HCl (1.8 M) was added until the equivalence point was approached (pH paper). The reaction mixture was stirred at room temperature for 6 h. The solid precipitate was filtered off and recrystallized from water to give compound **1** (Figure S1) in 80% yield, m. p. 180 °C (Lit. mp. 180 °C^29^.

##### Synthesis of 3-methyl-7-(phenylamino)-4*H*-[1,3,4] thiadiazolo[2,3-c][1,2,4] triazin-4-one (5)

**Fig. 2:**
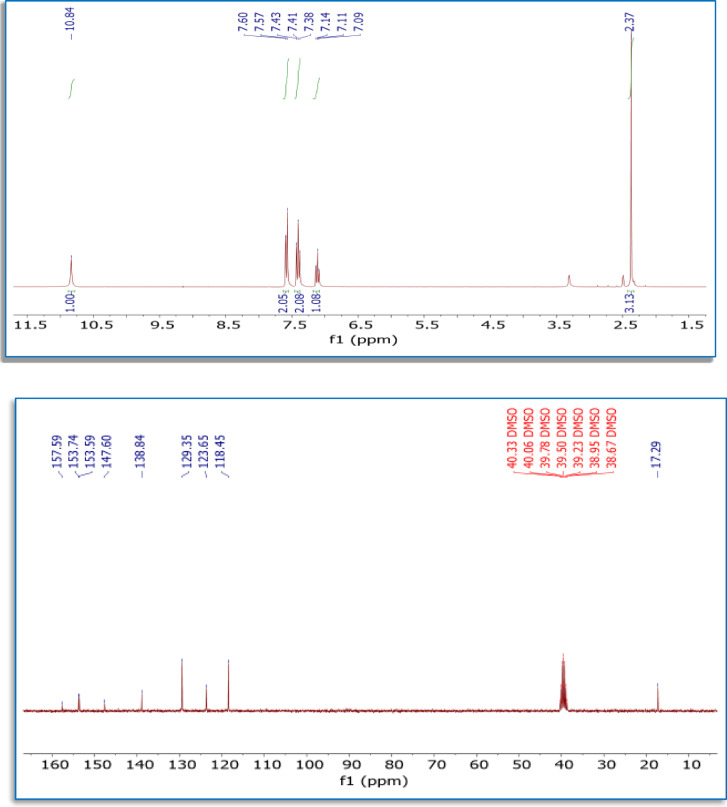
^1^H and ^13^C NMR spectra of compound **5**.

A mixture of 4-amino-6-methyl-3-thioxo-3,4-dihydro-1,2,4-triazin-5(2 H)-one (**1**) (1.58 g, 10 mmol) and phenyl isothiocyanate (1.35 g, 1.2 mL, 10 mmol) in EtOH (20 mL) was heated under reflux for 3 h, then allowed to cool to room temperature. After cooling, the precipitated solid was filtered off and dried. Re-crystallization from DMF/EtOH afforded the white product, yield (72%), m.p. 290–292 °C [Lit. m.p. 291-3^30^, IR (KBr) ν 3248 (NH), 1690 (C = O) cm^-1^; ^1^H NMR (DMSO-*d*_6_): δ 2.37 (s, 3 H, CH_3_), 7.09–7.60 (m, 5 H, ArH`s), 10.84 (s, 1H, NH); ^13^C NMR (DMSO-*d*_6_): δ 17.29, 118.45, 123.65, 129.35, 138.84, 147.60, 153.59, 153.74, 157.59 (C=O). ^1^H & ^13^C NMR charts of the ligand are presented in Fig. 2.

##### Synthesis of Pd(II)-complex

Synthesis of the Pd(II) complex was performed by reacting [PdCl_4_]^2−^ with the prepared compound **5**, dissolved in DMF (5 mL ), at a 1:1 molar ratio (Pd: L). The [PdCl_4_]^2−^ solution was obtained by heating KCl (2.0 mmol) and PdCl₂ (1.0 mmol) in distilled water (20 mL) at 70 °C under continuous stirring for 3 h. The resulting yellow precipitate was collected by filtration, washed thoroughly with absolute ethanol and distilled water, and then dried in an oven at approximately 70 °C. The final product was stored in a vacuum desiccator over P_2_O_5_. The reaction afforded a yield of 80.42%, anal. calcd. for **C**_**23**_**H**_**37**_**Cl**_**2**_**N**_**9**_**O**_**5**_**PdS** (728.99): C, 37.89; H, 5.12; N, 17.29; Found: C, 37.33; H, 5.11; N, 17.05. IR (KBr): ν 3250 (NH), 1650 (C = O) cm^−1^; ^1^H NMR (DMSO-*d*_6_): δ 2.38 (s, 3 H, CH_3_), 7.1–7.6 (m, 5 H), 10.84 (s, 1H, NH).

### Computational study

The input files for all compounds were prepared with GaussView 5.0.8. Gaussian 09 rev. A.02 software was used for all calculations using the DFT/B3LYP method. 6–311 + G(d, p) and LANL2DZ are the standard basis sets for the synthesized ligand and its Pd(II) complex, respectively^31,32^. A docking study was performed using MOE 2014.09 software. Regularization and optimization for protein and ligand were performed. Each docked compound was assigned a score according to its fit in the ligand binding pocket (LBP) and its binding mode.

### Biological activity

All general procedures regarding biological evaluations are discussed in detail in the ESI file.

#### Antimicrobial study

Antimicrobial susceptibility of the pathogenic selective strains was carried out by the agar well diffusion method^33^ against the target compounds, in parallel with Gentamicin, Ampicillin, and Nystatin as standard medications. The diameters of the zones of inhibition (ZOI, mm) were measured accurately to indicate antibacterial and antifungal potentials.

#### Cell viability assessment assay

The MTT method was employed to evaluate cell viability^34^.

#### Bovine serum albumin denaturation inhibition measurement

Screening for protein denaturation inhibitors is crucial in anti-inflammatory screening investigations. A protein denaturation test was performed as described by Gambhire et al.^35^ with slight modification.

## Results and discussion

### Synthetic route of ligand 5

A modified method has been developed for the synthesis of 4-amino-6-methyl-3-thioxo-3,4-dihydro-1,2,4-triazin-5(2 H)-one (**1**) from thiocarbohydrazide and sodium pyruvate by stirring at room temperature, rather than heating, which was considered a crucial step in this reaction^29^.

**Scheme 1:**
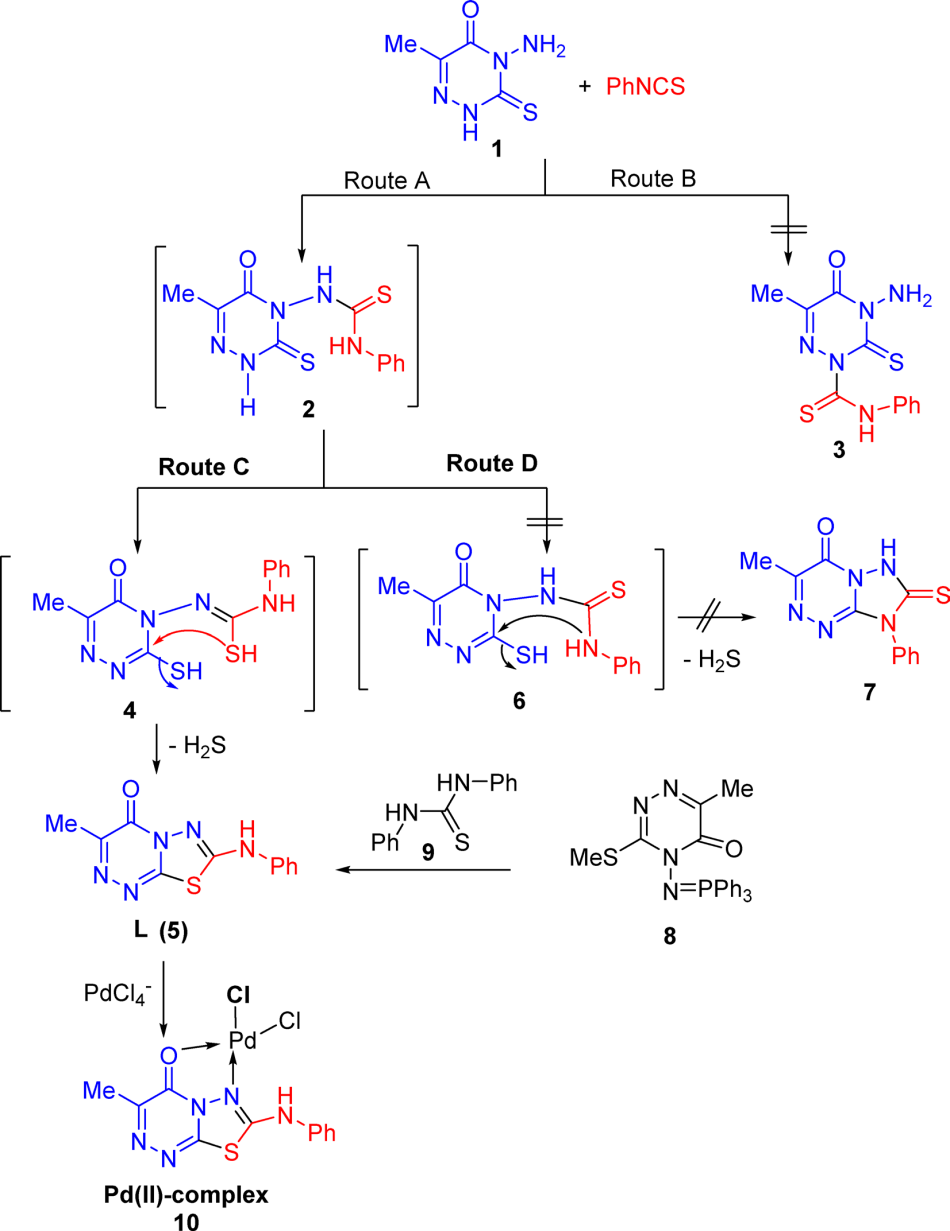
Regioselective synthesis of Pd(II)-complex (**10**).

Refluxing of product **1** with phenylisothiocyanate (PhNCS) in absolute ethanol for 6 h afforded 3-methyl-7-(phenylamino)-4 H-^1,3,4^ thiadiazolo[2,3-c]^1,2,4^ triazin-4-one (**5**) (Scheme 1). The isolated product **5** was identical in all respects to an authentic sample prepared previously by heating triazine **1** with PhNCS in DMF for 48 h^30^ or by heating 6-methyl-3-methylthio-4-triphenylphosphoranylideneamino-5-oxo-4,5-dihydro-1,2,4-triazine (**8**) with *N*,* N’*-diphenyl thiourea (**9**) in dry benzene for 14 h^36^. The chemical structure of thiadiazolotriazine **5** was validated and confirmed using spectral data (IR, NMR) and elemental analyses.

As outlined in Scheme 1, the reaction of triazine **1** with PhNCS proceeded in a regioselective manner. It can start via nucleophilic attack by the 1st nitrogen (route **A)** or 2nd nitrogen (route B) to the thiocarbonyl carbon of PhNCS. The distinction between the two possible routes can be made easily using the spectral data of an isolated product. For example, in the IR spectrum of the isolated product, the absence of the NH_2_ absorption bands excluded structure 3 (route B). Also, the presence of only one absorption peak at 3248 cm^−1^ due to the NH function excluded structures 2 (route A) and **3**, and suggested another structure 5 (route C) or **7** (route D) for the reaction product. In addition, the absence of an absorption peak due to the thiocarbonyl function in the IR spectrum of the isolated product excluded structure 7. Other evidence, confirming structure 5 and excluding structure 7, came from NMR spectral analysis of the isolated product. For example, the proton NMR spectrum of the reaction product showed a singlet signal at δ 10.84 ppm due to the NH proton; the ^13^C-NMR spectrum also revealed the absence of any signal for the thiocarbonyl carbon around δ 179.00 ppm^37^. The ^1^H &^13^C NMR spectra are presented in Fig. 2.

### Structural characterization of the ligand (5) and its Pd(II)-complex (10)

#### The physical properties and spectral data

The synthesized Pd(II)-complex (**10**) is stable at ambient temperature. Based on the elemental analysis and spectral studies, the coordination geometry has been assigned as shown in Scheme 1. The complex can be symbolized as [Pd(L)(Cl)_2_].4DMF. The molar conductance in 10^− 3^ molar DMSO solution is in the range 5.5–8.8 ohm^− 1^ cm^2^ mol^− 1^, which reveals the non-electrolytic environment of the palladium(II) complex^38–40^ with the direct covalent binding between the Pd(II) and the donor sites of the ligand. The results of the elemental microanalyses are in respectable agreement with the suggested formula (Scheme 1). The CHN analyses and the sensible features of the successfully prepared compounds are recorded in Table 1.

**Table 1:** Some of the physical properties of the prepared compounds.

Compd molecular formula	Mol. Weight g.mol^− 1^	Color	Yield %	Found (Calc.) %		
				C	H	*N*
C_11_H_9_N_5_OS (5)	259.29	Pale yellow	72	50.20 (50.96)	3.33 (3.50)	26.71 (27.01)
[Pd(C_11_H_9_N_5_OS)Cl_2_].4DMF	728.99	Yellow	80.42	37.33 (37.89)	5.11 (5.12)	17.05 (17.29)

The vibration modes of ligand **5** and its Pd(II) complex are represented in Table 2 and Figure S2. A broad absorption peak for the υ(N–H) stretching vibration is observed in the range of (3248–3250) cm^−1^^41^. This vibration remains almost unperturbed in the coordination environment, signifying the non-participation of the NH moiety in the coordination mechanism, in agreement with other investigations^42^. Stretching frequencies around 1690 cm^−1^ and 1550 cm^−1^ are assignable to the υ(C = O) of 1,2,4-triazine, and υ(C = N) of thiadiazole ring segments^43^, respectively were shifted to 1650 cm^−1^ and 1566 cm^−1^ in complexation with Pd(II) ion, demonstrating the contribution of the thiadiazole nitrogen and carbonyl oxygen in chelation, compatible with assignments of additional workers^44,45^. The significant red shift in the carbonyl frequency (Δν = −39 cm^−1^) accompanied by a blue shift in the azomethine band (Δν = +16 cm^−1^) offers quantitative confirmation of the ligand’s bidentate coordination via the carbonyl oxygen of the triazine ring and the thiadiazole nitrogen. Assignments of the proposed coordination positions are further supported by the appearance of medium bands in the metal complex at 378 cm^−1^ and 509 cm^−1^, which could be assigned to the coordinated υ(Pd–O) and υ(Pd–N)^25^ vibrations, respectively. An additional weak band was observed around 285 cm^−1^, which can be assigned to υ(Pd–Cl)^38^. The above interpretation is completed by a vibrational assignment at 694, 756, 1095, and 1327 cm^−1^ that may be ascribed to *v*(NCO bend + CN str), *v*(CN sym str), *v*(CH_3_ rock), and *v*(NCH+ CH_3_), which match the free (non-coordinated) DMF molecules^46^. The DMF carbonyl did not display a significant coordination-induced red shift. This proves that the coordination sites are occupied by the ligand and chloride ions, leaving DMF as a lattice occupant. The vibrational bands around 1489 cm^−1^ and 840 cm^−1^ were ascribed to the vibrational stretches of the υ(N-N) and υ(C-N) of the 1,2,4-triazine moiety, in agreement with the designated values of 1442 cm^−1^ and 827 cm^−1^ region in similar works^47–49^. The Pd(II) complex assembly did not result in a change to a lower wave number, indicating that the triazine nitrogen was not a factor in the chelation process.

**Table 2:** Comparison between the experimental and theoretical vibrational frequencies (cm^−1^) for ligand (**5**) and its metal complex (**10**).

Compds	ν(C = *N*) + ν(C-S) thiadiazole ring		ν(C = O, C-*N*, *N*-*N*) 1,2,4-triazine ring		ν(Pd-O)		ν(Pd-*N*)		ν(Pd-Cl)	
	DFT	Exp.	DFT	Exp.	DFT	Exp.	DFT	Exp.	DFT	Exp.
L (5)	1552, 760	1550, 760	1660, 840, 1489	1689, 1489, 840	–	–	-	–	–	–
Pd-L (10)	1560, 760	1566, 760	1680, 1488, 824,	1650, 1496, 830	376	378	504	509	280	285

**Fig. 3:**
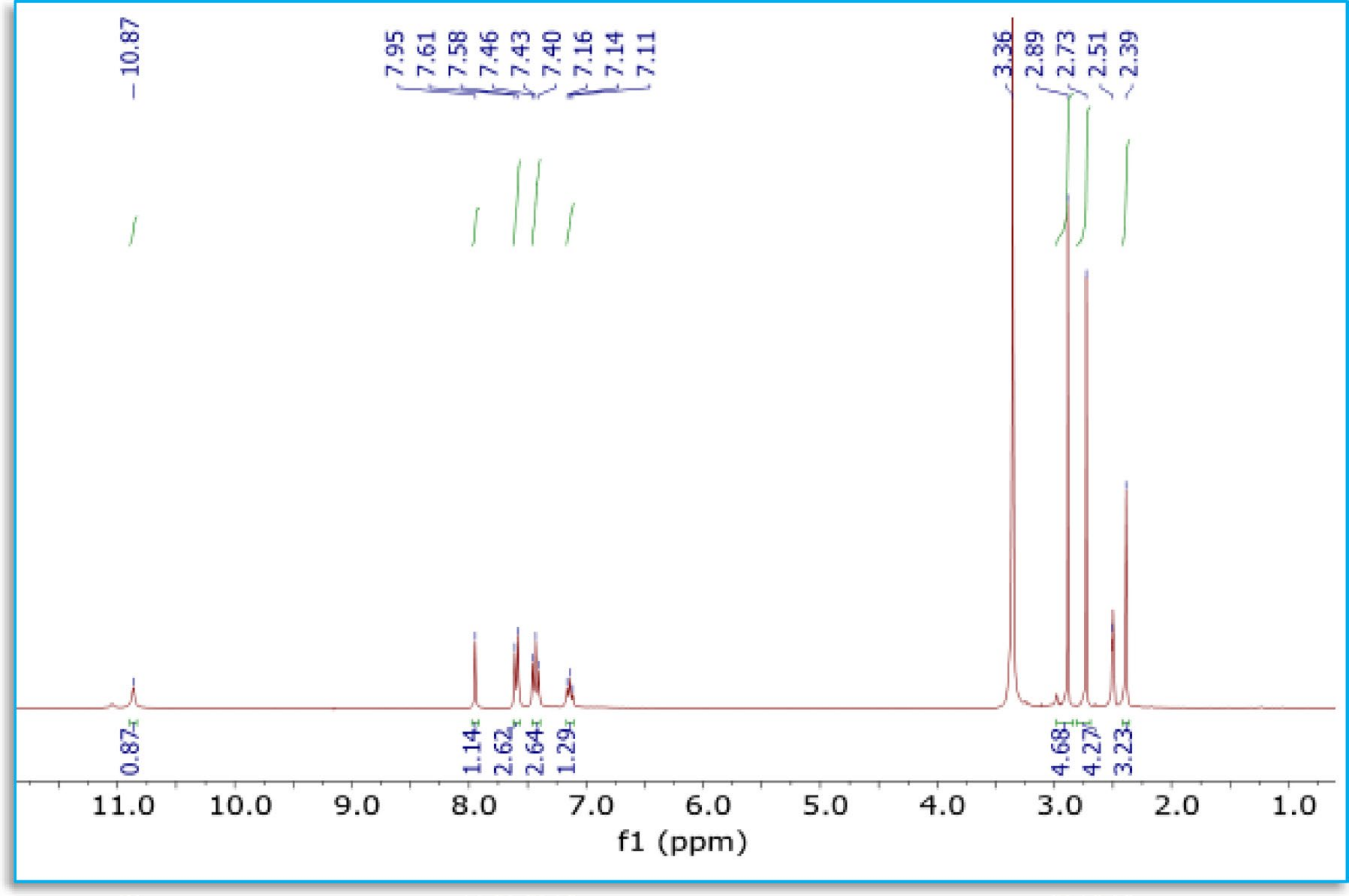
^1^H NMR spectrum of Pd(II) complex.

^1^H-NMR spectrum of the reaction product **(5)** (Fig. 2; Table 3) reveals a singlet signal at δ 10.84 ppm corresponding to the NH proton^48,50^. Additionally, the signals corresponding to the protons of phenyl and methyl substituents are seen at δ (7.09–7.60) ppm and 2.37 ppm, respectively^51^. The three protons on the phenyl ring exhibit distinct chemical shifts, giving rise to distinct peaks. This discrepancy arises from its proximity to the electron-withdrawing nitrogen atom. The remaining peaks correspond to the other three protons in this system. Upon complexation, the aromatic *H*_*a*_ protons shift downfield from 7.09 to 7.60 ppm in the free ligand to 7.11–7.95 ppm in the Pd(II) complex because coordination of the C = N group to Pd(II) withdraws electron density from the ligand, deshielding nearby protons. The *H*_*b*_ and *H*_*c*_ peaks also move slightly (*H*_*b*_: 10.84 → 10.87 ppm; *H*_*c*_: 2.37 → 2.39 ppm), and new DMF signals at 3.31 ppm appear, consistent with changes in the electronic environment upon binding as summarized in Table 3; Fig. 3. Moreover, the ^13^C NMR spectrum of non-binding triazine compound (**5**) presented (Table 4; Fig. 2) a peak at δ 153.74 ppm belong to the carbon of (***C***H = N) in thiadiazole ring, then peaks at the range δ (118.45-138.84) ppm were attained to an aromatic carbon, as well as, peaks at δ 157.59 ppm and 147.60 ppm referred to δ (C = O) and δ (C = N) carbons, respectively, of triazine ring. As well, the ^13^C-NMR spectrum did not show any signal in the region around δ 179.00 ppm, indicating the absence of thiocarbonyl carbon (**C** = S)^37^. The ^1^H NMR spectrum analysis of the Pd(II) complex reveals a shift in the aromatic protons attributable to chelation, as seen in Fig. 3. The emergence of the NH proton signal at a magnitude comparable to that of the ligand upon chelation suggests that it does not participate in the coordination center.

**Table 3:** Analyses of the ^1^H-NMR spectra of the ligand (**5**) and its Pd(II) complex.


	H^a^		H^b^		H^c^		DMF protons	
	Exp.	DFT	Exp.	DFT	Exp.	DFT	Exp.	DFT
L (5)	7.09–7.60	7.20–7.60	10.84	6.25	2.37	2.20	–	–
Pd(II) complex	7.11–7.95	6.85–7.20	10.87	6.50	2.39	1.70–1.50	3.31	5.10

**Table 4:** ^13^C NMR data of theoretical investigations based on ligand (**5**).

Compd	C = *N* (thiadiazole ring)		C = *N* (triazine ring)		C = C (aromatic ring)		C = O (triazine)	
L (5)	Theo.	Exp.	Theo.	Exp.	Theo.	Exp.	Theo.	Exp.
	159	153.58	148	147.58	120–138	118.45-138.84	157	157

#### Electronic transitions and magnetic studies

**Fig. 4:**
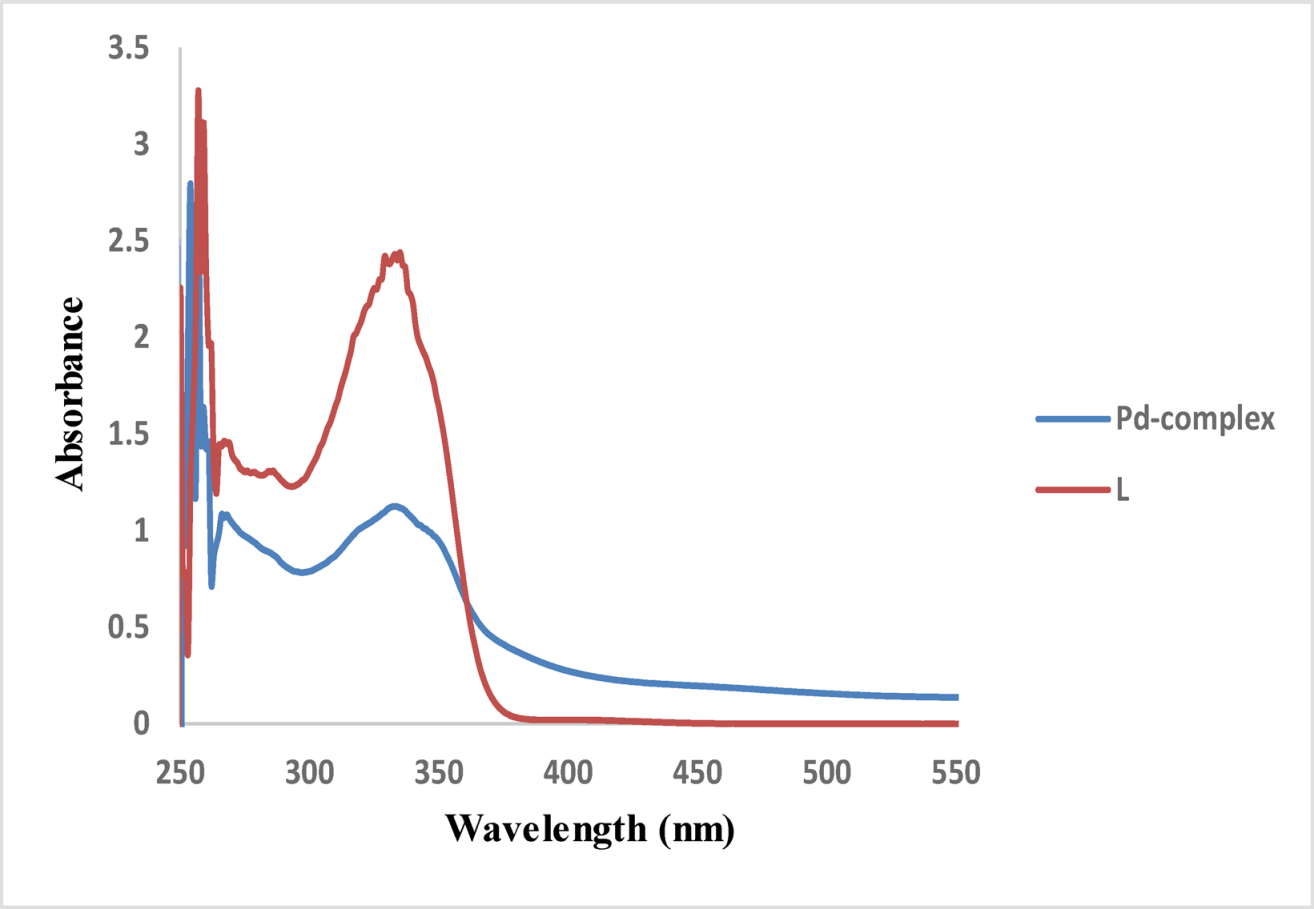
Uv-vis spectra of compound (**5**) and its **Pd(II)** complex.

The electronic transitions spectrum data for the free ligand (**5**) in DMF displays four distinct bands in the ranges (259, 268, 337) nm. The higher energy bands unequivocally originate from the aromatic π→π* transitions of the ligand L. The medium-energy band is a result of composite bands arising from charge transfer (CT) transitions. The lower energy band is definitely ascribed to the n→π* transitions of the azomethine groups. The ligand transitions align well with those of analogous compounds previously documented in the literature^52^. The spectrum of the Palladium (II) complex exhibits these absorptions but shifted to (257, 264, 335) nm accordingly, therefore corroborating the coordination of the ligand with metallic ions^53,54^. The electronic transitions are seen in Fig. 4; Table 5.

**Table 5:** Electronic spectral data for ligand **5** and its Pd (II) complex.

Compound	(λ_max_) nm	(*ύ* ) cm^− 1^	µ_eff_		Assignment
			Theo.	Exp.	
L (5)	259	38,610			π → π* of the triazine ring
	268	37,313			n → π* transitions involving the triazine, phenyl rings, and/or azomethine groups
	337	29,674			n→π* transitions of the azomethine groups
Pd(II) complex	257	38,911	0.0	0.0	π → π* of the triazine ring
	264	37,878			n → π* transitions involving the triazine, phenyl rings, and/or azomethine groups
	335	29,851			Mixed LLCT/LMCT contributions

LLCT, Ligand-to-Ligand Charge Transfer and LMCT, ligand-to-metal charge transfer.

#### Thermal stability inspection of Palladium(II) complex

**Fig. 5:**
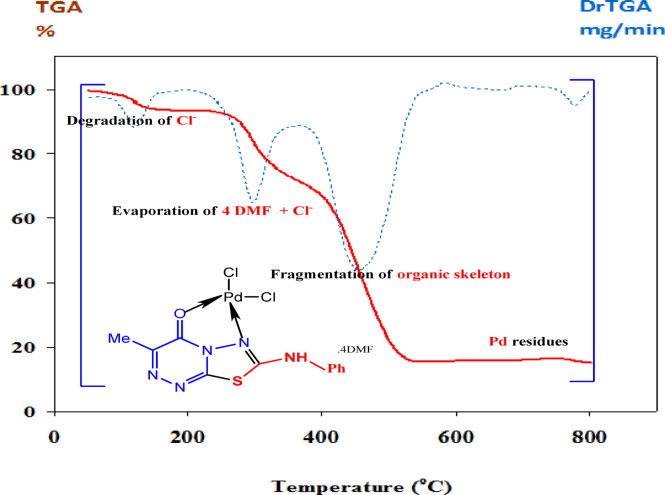
TGA curve of **Pd(II)** complex.

Thermogravimetric analysis (TGA) is a crucial instrumental technique for detecting thermal changes as temperature increases. The decomposition pattern of the complex has been explained by the reactions shown in Fig. 5, and the decomposition stages, temperature range, degradation product loss, and the found and calculated weight loss percentages of the complexes are given in Table 6. The TGA/DTA curve for the synthesized Pd (II) complex was carried out, and the first decomposition step at a temperature lower than 126 °C (obs = 4.70%, calc = 4.86%) is assignable to the elimination of one chloride ion as a HCl molecule from the inner sphere of the coordination entity. The subsequent decomposition stage observed within the temperature range 126–300 °C is attributed to the evaporation of DMF solvent molecules and the remaining chloride ion, with continuous fragmentation of the organic skeleton at 469 °C (obs. = 80.0%, calc. = 80.53%). The evolution of DMF molecules at a moderate temperature range (beginning at ~ 126 °C) before the decomposition of the metal–ligand framework, suggests that these molecules are present as lattice-held solvent rather than being directly coordinated within the inner coordination sphere. The thermal degradation of the palladium (II) complex molecule ended with a final stable residue, as identified from the mass loss consideration, which is Pd metal (obs. = 15.30%, calc. = 14.59%). The computed thermal degradation data in Table 6 are in good agreement with the suggested microanalytical data in Table 1.

**Table 6:** Thermal decomposition pattern of the Pd(II) complex.

Compound	Temp. range / ^o^C TGA	Wt. loss %, Calc. (F.)	Assignments
**[Pd(C**_**11**_ **H**_**9**_ **N**_**5**_ **OS)Cl**_**2**_ **.4DMF]**	below 126 126–300 469	5.0 (4.70) 80.53 (80.0) 27.25 (27.9)	HCl 4DMF + HCl C_11_H_7_N_5_OS
**Residue**	> 540	14.59 (15.30)	Metallic Pd

#### Mass spectrometry of the Pd(II)-complex

**Fig. 6:**
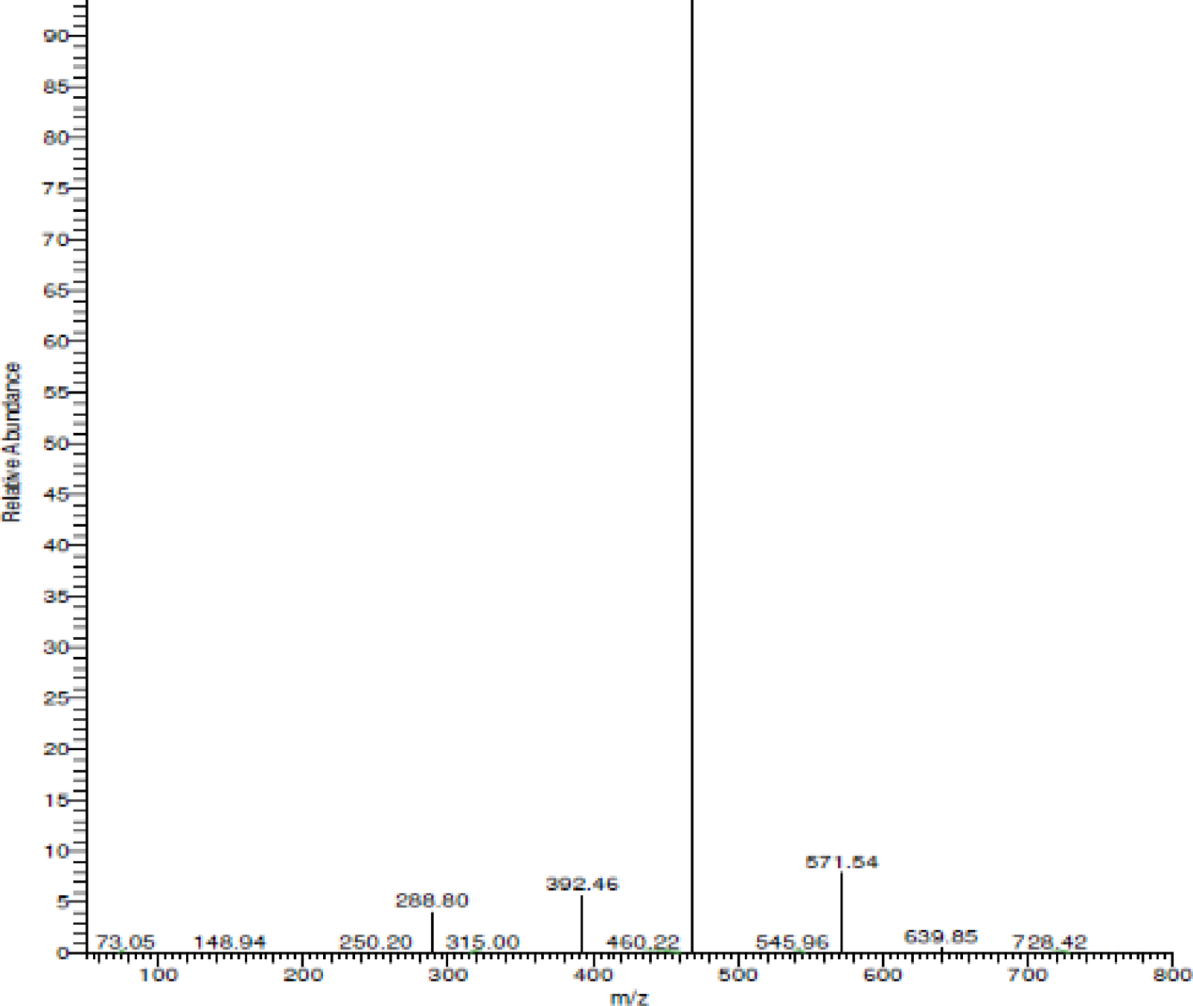
Mass spectrometry chart of the Pd(II)-complex.

The purity of the ligand and its palladium complex is confirmed using electron impact mass spectrometry (Fig. 6). The mass spectrometric investigation of the Pd(II) complex (M. wt = 728.99 g/mol) provides critical insights into its structural integrity and the nature of its coordination sphere. The molecular ion peak observed at m/z **=** 728.42 (Calculated m/z = 728) corresponds to the intact complex associated with four N, N-dimethylformamide (DMF) solvent molecules. The fragmentation hierarchy initiates with the sequential elimination of loosely bound solvent species. A distinctive fragment at m/z **=** 640.86 consequences from the damage of one DMF molecule and a methyl group (total mass loss of 88.12 g/mol). This is followed by a significant transition at m/z **=** 571.54 (Calculated m/z = 569.86 ≈ 570), which is attributed to the elimination of the coordination chloride portions, marking the onset of core structure fragmentation after partial solvent removal. As the fragmentation proceeds, the stepwise decomposition of the organic ligand framework becomes evident. The peak at m/z **=** 460.22 (Calculated m/z = 460.45) indicates the loss of an additional DMF molecule and the partial fragmentation of the phenyl ring. Subsequent cleavage of a C_4_H_4_O moiety leads to the fragment at m/z **=** 392.46 (Calculated m/z = 392.22), corresponding to the breakdown of the aromatic/heterocyclic ring system. The final stages of decomposition involve the loss of the remaining DMF and NH-oxygen units to yield a peak at m/z **=** 288.80 (Calculated m/z = 288.77), eventually resulting in the stable Pd-containing fragment at m/z **=** 148.94 (Calculated m/z = 148.74) after the expulsion of the C_4_H_4_N_4_S heterocyclic core. These findings unequivocally support the proposed coordination geometry and the presence of both inner-sphere chloride ligands and outer-sphere solvent molecules. The suggested fragments are seen in Scheme S1 and Table S1.

#### X-ray diffraction of the Pd(II)-complex

**Fig. 7:**
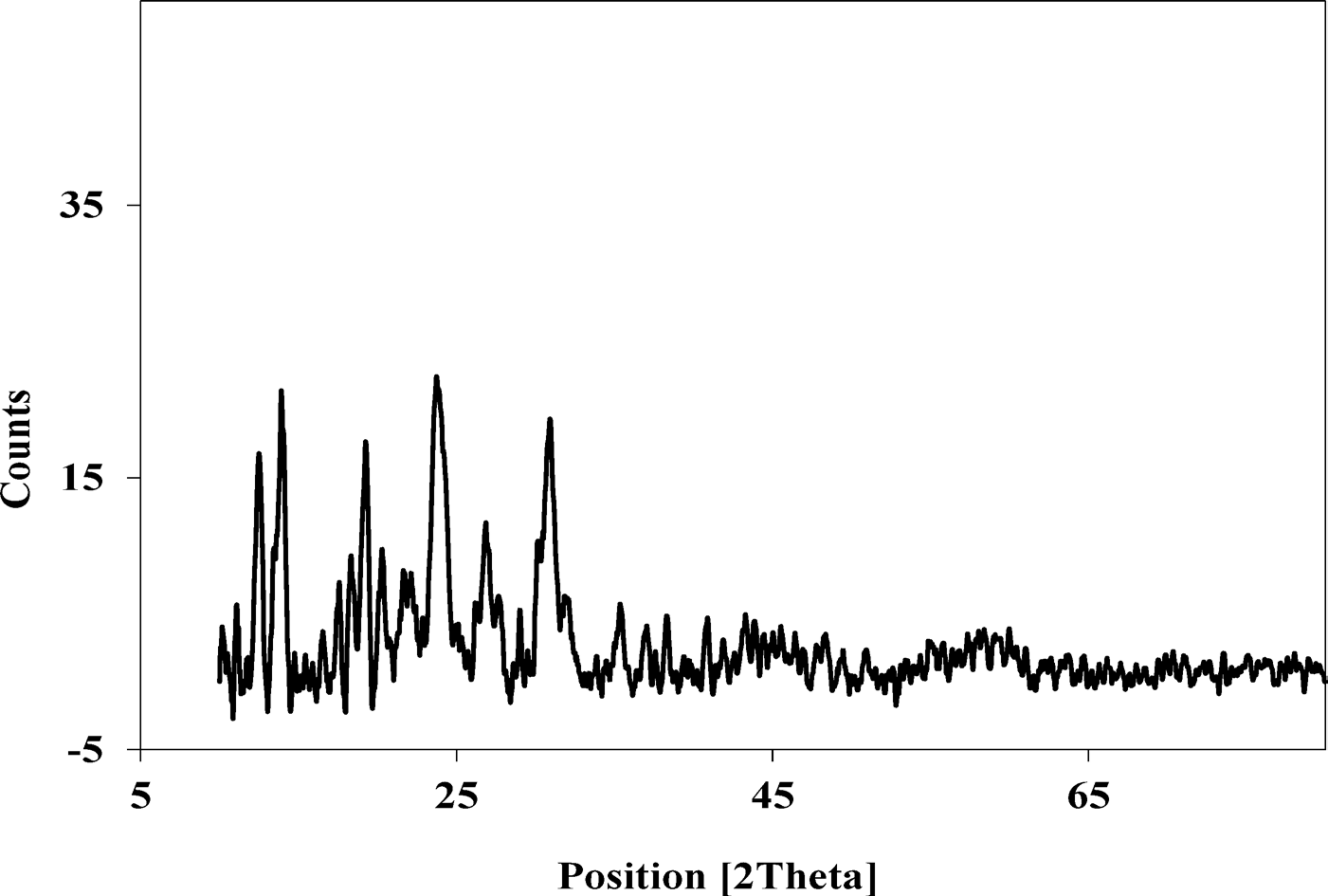
XRD patterns of Pd(II)-complex.

The XRD diffraction pattern of the solid Pd(II) complex is displayed in Fig. 7. The XRD pattern shows the sharp crystalline peaks, indicating its crystalline phase. The major refluxes were measured, and the corresponding d-spacing values are represented in Table 7. The average crystallite size of the complex was calculated using Scherer’s formula^55,56^. The particle size is given by t = 0.9 λ/Bcosθ, where B is the half-width (in radians), t is the crystal thickness (in nm), λ is the wavelength, and θ is the Bragg angle. The particle size corresponding to each diffraction maximum for the crystalline compounds is determined from the measurement of the half-width of the diffraction peak. The calculated average size of the Pd(II) complex is 36.49 nm. The value of the crystallinity index (CI) was determined as 66.37% using the equation CrI=A_crystal_/A_total_, where A_crystal_ and A_total_ represent the diffraction peak areas of the crystalline region and the total region, respectively.

**Table 7:** The major refluxes and the corresponding d values of the Pd(II) complex.

2θ(_Obs_) °	d(_Obs_) Å	2θ(_Obs_) °	d(_Obs_) Å
12.370	7.14962	21.605	4.10990
13.895	6.36825	23.583	3.76943
17.486	5.06777	26.741	3.33106
18.203	4.86976	27.586	3.23091
19.120	4.63800	30.764	2.90403

### DFT study

#### Optimization, Frontier molecular orbitals, and molecular electrostatic potential

**Fig. 8:**
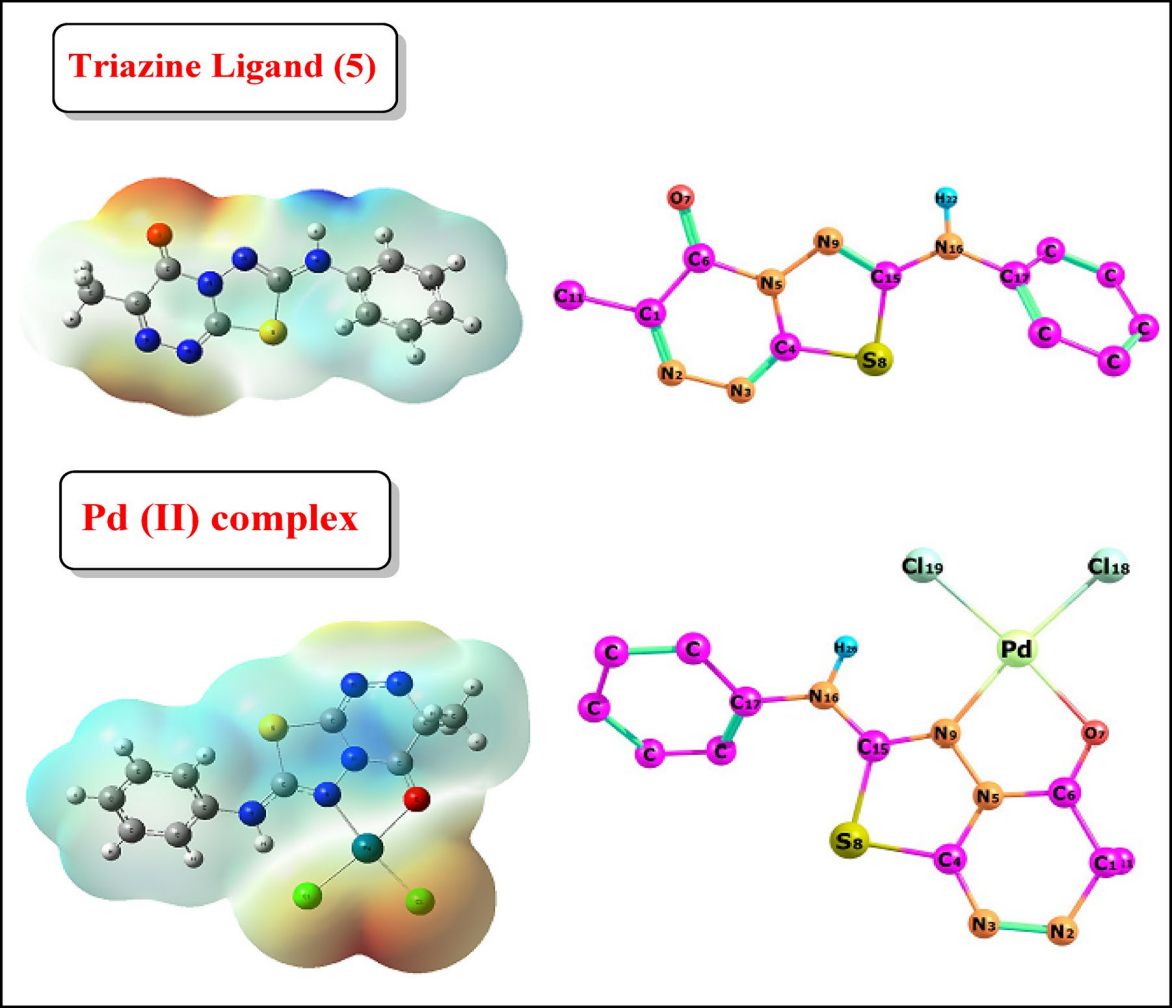
The optimized structures and molecular electrostatic potentials (MEPs) of the triazine ligand (**5**) and its coordinated Pd-complex.

The ligand **5** was proposed to be formed through the formation of compounds (**1–4**). All suggested structures are optimized, and ground-state properties are calculated to compare all suggested compounds. The optimized structures and ground-state properties are represented in Fig. 8; Table 8. The chemical reactivity and stability of molecules are represented by the energy gap (E_HOMO_–E_LUMO_)^57^. The negative magnitude of E_HOMO_ and E_LUMO_ establishes the stability of compounds^58^. Compound **5** had the largest energy gap, together with a significantly negative optimized energy value. The significant energy gap indicates excellent stability, therefore favoring the production of compound **5**, which was experimentally isolated. The reactivity order is 2 > 3 > 1 > 7 > 5. Following complexation, it was determined that compound **5** was transformed into a Pd(II) complex exhibiting enhanced reactivity. The improvement in biological characteristics is expected to occur with increased reactivity of the Pd complex form. From the data in Table 8, the more nucleophilic character was observed with the low electrophilicity index (ω) value; the electrophilicity order is 2 > 7 > 3 > 1 > 5. Therefore, compound **5** was the highest nucleophilic ligand. From the optimization energy, gap energy, and ω values, it may be concluded that compound **5** was the favored and most easily separable compound.

**Fig. 9:**
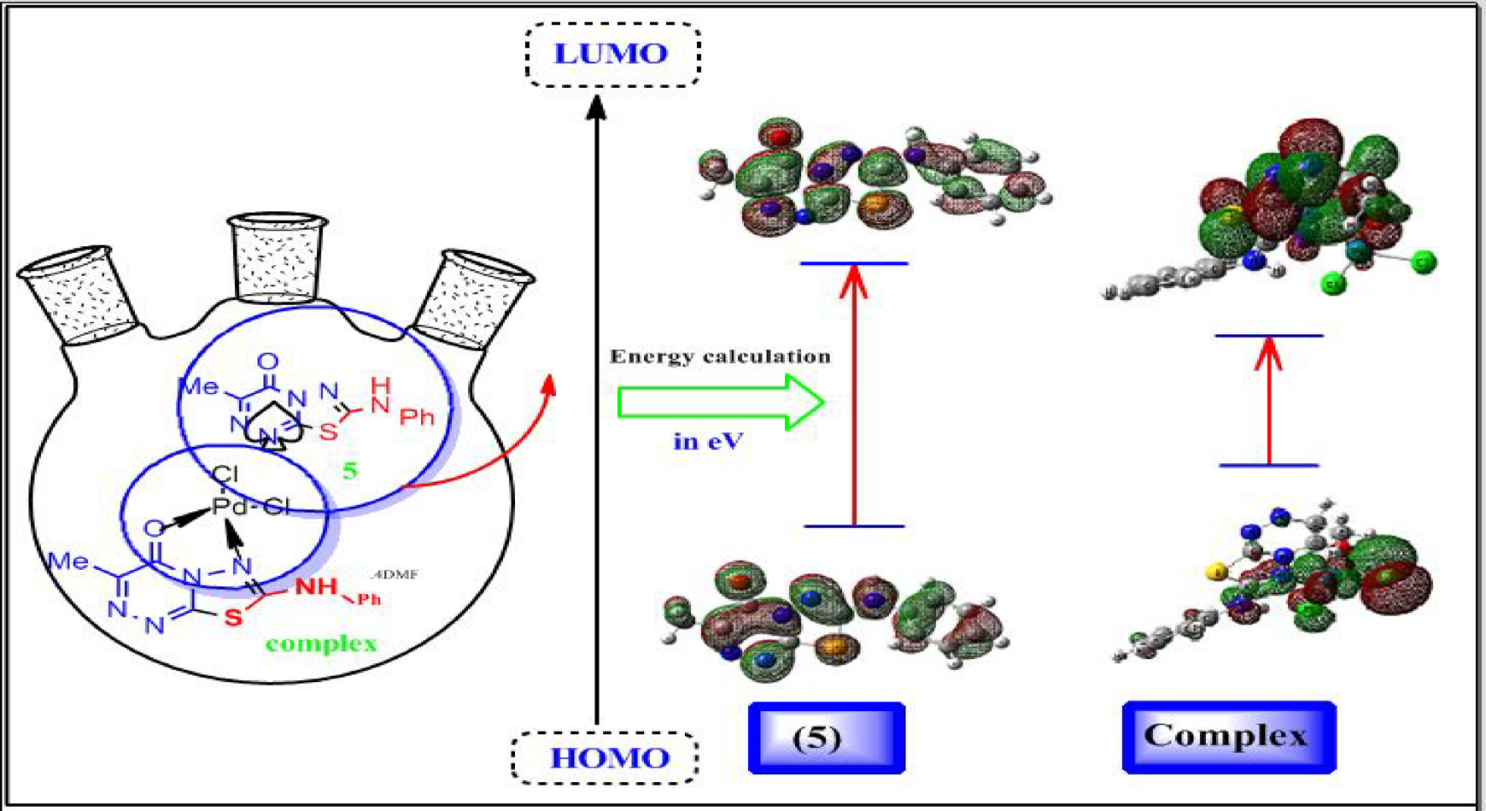
Molecular orbitals and the respective energy gap of the ligand **5** and its Pd(II) complex.

**Fig. 10:**
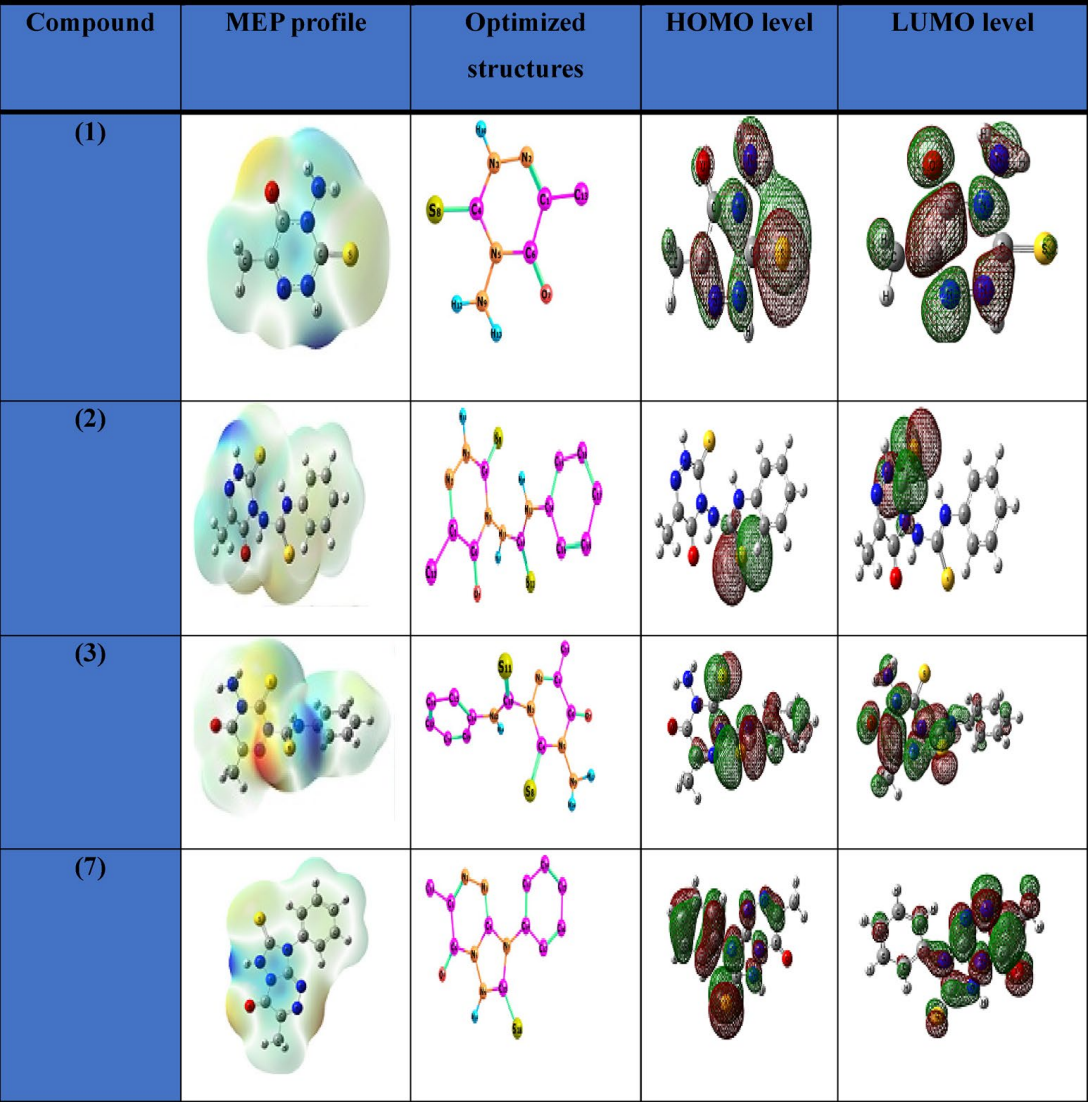
The molecular electrostatic potentials (MEPs) profile, optimized structures, and the respective energy gap of the intermediate (**1**, **2**, **3**, and **7**) structures.

The HOMO and LUMO electron cloud distribution diagrams are shown in Figs. 9 and 10, respectively. The palladium metal ion is coordinated to four atoms via the **N9** and **O7** donor sites of ligand **5**, and its coordination is completed and neutralized by two chloride ions. The optimized angles are around 90^◦^ as **[**(C119-N9-O7) = 97.118^◦^ and (C113-Pd-N9) **=** 95.566^◦^**]** which support the suggested square-planar structure. Some bond lengths are lengthened and sometimes are shortened to optimize the coordination sphere, as seen in Table 9; Fig. 9. The molecular electrostatic potential (MEP) designates the red zone as the region of the highest electron density. In contrast, the blue zone represents the lowest, as seen in Fig. 10. The red color was distributed around N9 and O7 donor sites with natural population charge values (-0.315) and (-0.602), respectively. The remaining part of the ligand **5** observed the highest positive electrostatic potential with an increase in the electron density of the Pd metal ion to become (0.198), and the charges of all elements are estimated and tabulated in Table 10.

**Table 8:** Ground state properties of the ligand and its metal complex using B3LYP/6-311G and B3LYP/LANL2DZ, respectively.

Parameter	1	2	3	5	7	Pd-complex
E_T_, Hartree	-848.58	-1571.38	-1571.37	-1171.93	-1171.64	-940.75
E_HOMO_, eV	-6.72	-5.57	-6.52	-6.52	-6.84	-3.92
E_LUMO_, eV	-2.36	-2.91	-2.41	-2.06	-2.43	-3.27
ΔE, eV	4.35	2.65	4.11	4.46	4.40	0.65
I=- E_HOMO_, eV	6.72	5.57	6.52	6.52	6.84	3.92
A= - E_LUMO_, eV	2.36	2.91	2.41	2.06	2.43	3.27
χ, eV	4.54	4.24	4.46	4.29	4.63	3.60
η, eV	2.17	1.32	2.05	2.23	2.20	0.32
S, eV^− 1^	0.22	0.37	0.24	0.22	0.22	1.52
µ, eV	-4.54	-4.24	-4.46	-2.27	-4.63	-3.60
ω, eV	4.74	6.78	4.85	4.13	4.88	19.71
ΔN_max_, eV	2.08	3.19705	2.17	1.925592	2.106977	10.95
Dipole Moment (Debye)	2.28	5.5037	2.92	6.4541	0.9172	10.25

**Table 9:** Some of the optimized bond lengths, Å, and bond angles, degrees, for ligand **5** and its Pd(II) complex using B3LYP/6-311G and B3LYP/LANL2DZ, respectively.

Bond length (Å)	1	2	3	5	7	Pd
Pd-O7	–	–	–	–	–	2.16543
Pd-N9	–	–	–	–	–	2.11216
Pd-Cl18	–	–	–	–	–	2.33582
R(C1-N2)	1.29527	1.29056	1.29246	1.30508	1.30872	1.52594
R(N2-N3)	1.34465	1.34973	1.35472	1.37124	1.36362	1.35982
R(N3-C4)	1.37075	1.36851	1.38289	1.29224	1.30356	1.32512
R(C4-N5)	1.37671	1.38994	1.38013	1.37227	1.36014	1.40120
R(N5-C6)	1.40203	1.42121	1.39856	1.41306	1.39738	1.37346
R(C6-O7)	1.21882	1.20890	1.21914	1.21139	1.21808	1.26896
R(C4-S8)	1.66583	1.66232	1.66646	1.76847		1.81762
R(N5-N9)	1.39684	1.40382	1.39813	1.37384	1.36049	1.41318
R(N3-C10)		1.45982	1.47318	–	–	–
R(N9-C10)		1.41607	–	–	–	–
R(C10-S11)	–	1.65767	1.64007	–	–	–
R(C10-N12)	–	1.35696	1.34759	–	–	–
R(N12-C14)	–	1.41895	1.41352	–	–	–
R(N9-C15)	–	–	–	1.29717	1.36438	1.33680
R(C15-N16)	–	–	–	1.36000		1.33873
R(N16-C17)	–	–	–	1.41624	–	1.43561
R(C15-S18)	–	–	–	–	1.65680	–
R(N7-C19)	–	–	–	–	1.45371	–
A(C119-N9-O7)	–	–	–	–	–	97.118
A(C113-Pd-N9)						95.566
A(C10-N12-C14)	–	128.420	132.519		–	83.406
A(N5-N9-C15)	–	–		109.743	110.997	79.406
A(C4-S8-C15)	–	–	–	87.222	–	94.410
A(C1-N2-N3)	117.887	118.071	119.092	121.141	123.282	121.207
A(N2-N3-C4)	128.078	128.711	126.111	116.771	116.883	114.806
A(N3-C4-N5)	112.885	112.881	113.226	126.468	122.278	126.962
A( C4-N5-C6)	124.530	123.546	125.173	120.260	125.311	127.195
A(N5-C6-C7)	114.300	121.082	120.709	123.053	120.807	126.660
A(N2-C13)	114.300	119.954	119.381	118.577	118.464	122.804
A( N3-C4-S8)	122.584	121.179	131.657	124.441	–	130.549
A(C4-N5-N9)	119.213	119.587	119.030	117.647	108.891	–
A(N5-C6-O7)	120.451	121.082	120.709	123.053	120.807	–
A(O3-N10-S11)	–	–	119.041	–	–	–
A(S11-C110-N12)	–	128.652	131.393	–	–	–
A(C15-N16-C17)	–	–	–	128.446	–	178.703
A(N9-C15-N237)	–	–	–	–	104.897	–
A(S18-C15-N7)	–	–	–	–	134.039	103.076
A(C15-N7-C19)	–	–	–	–	127.497	–

**Table 10:** MPA(NPA) for ligand **5**, intermediates and Pd(II) complex using B3LYP/6-311G and B3LYP/LANL2DZ, respectively.

Element	1	2	3	5	7	Pd-complex
Pd	**–**	**–**	–	–	–	0.134 (0.198)
C1	-0.627 (-0.692)	-0.133 (0.148)	0.063 (0.154)	0.211 (0.138)	0.035222 (0.13194)	-0.309 (-0.089)
N2	0.202 (0.322)	0.098 (-0.203)	0.147 (-0.203)	-0.042 (-0.234)	0.239477 (-0.21513)	-0.042 (0.225)
N3	0.134 (-0.363)	-0.218 (-0.385)	0.477 (-0.324)	-0.088 (-0.338)	-0.245775 (-0.34844)	-0.005 (-0.211)
C4	-0.556 (-0.599)	0.096 (0.245)	-0.290 (0.241)	-0.095 (0.264)	-0.20520 (0.60623)	-0.131 (0.267)
N5	0.575 (0.432)	0.420 (-0.323)	0.226 (-0.284)	0.124 (-0.286)	-0.034549 (-0.24374)	-0.179 (-0.131)
C6	-0.642 (-0.262)	0.218 (0.623)	0.288 (0.597)	0.239 (0.608)	0.259010 (0.59758)	0.414 (0.371)
O7	-0.161 (0.031)	-0.251 (-0.54)	-0.350 (-0.602)	-0.314 (-0.569)	-0.338372 (-0.60215)	0.367 (-0.255)
S8	-0.375 (-0.468)	-0.538 (-0.138)	-0.455 (-0.155)	0.119 (0.377)	–	0.367 (0.223)
N9	-0.040 (0.114)	–	-0.260 (-0.390)	0.049 (-0.315)	-0.012417 (-0.39023)	-0.170 (-0.188)
S11	–	-0.470 (-0.140)	-0.310 (-0.029)	–	–	–
N12	–	-0.004 (-0.456)	-0.007 (-0.550)	–	–	–
C15	-0.043 (-0.139)	–	–	–	0.387804 (0.24040)	-0.018 (0.186)
N16	-1.147 (-1.244)	**–**	**–**	0.0180 (-0.607)	–	-0.346 (-0.308)
C17	**–**	**–**	**–**	-0.036 (0.142)	–	-0.236 (0.075)
Cl18	-0.608 (-0.732)	**–**	**–**	–	–	-0.210 (-0.187)
S18	**–**	**–**	**–**	–	-0.648 (-0.118)	**–**

### Antimicrobial activity with molecular docking

**Fig. 11:**
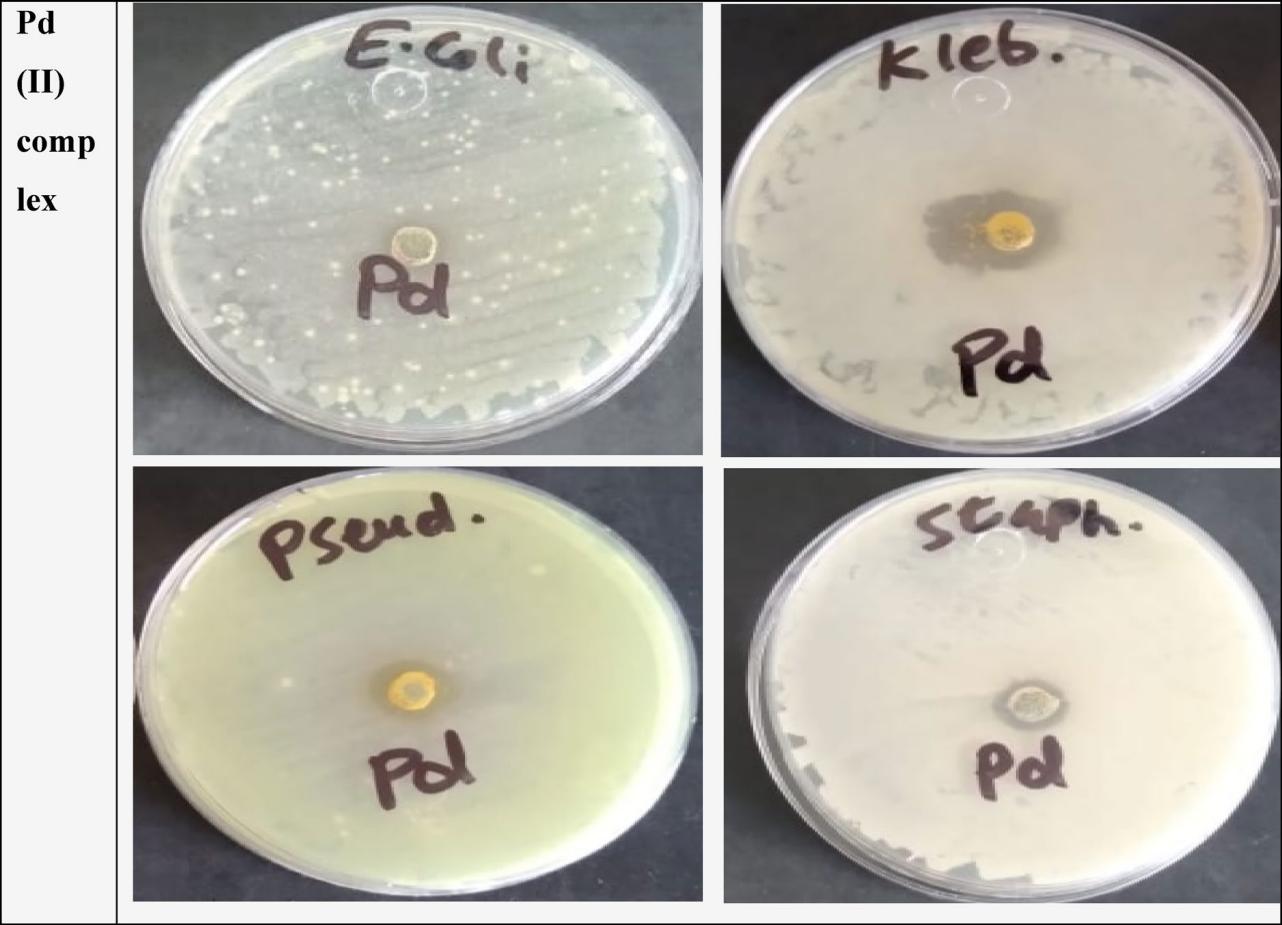
Some plates pictures of the antibacterial.

**Fig. 12:**
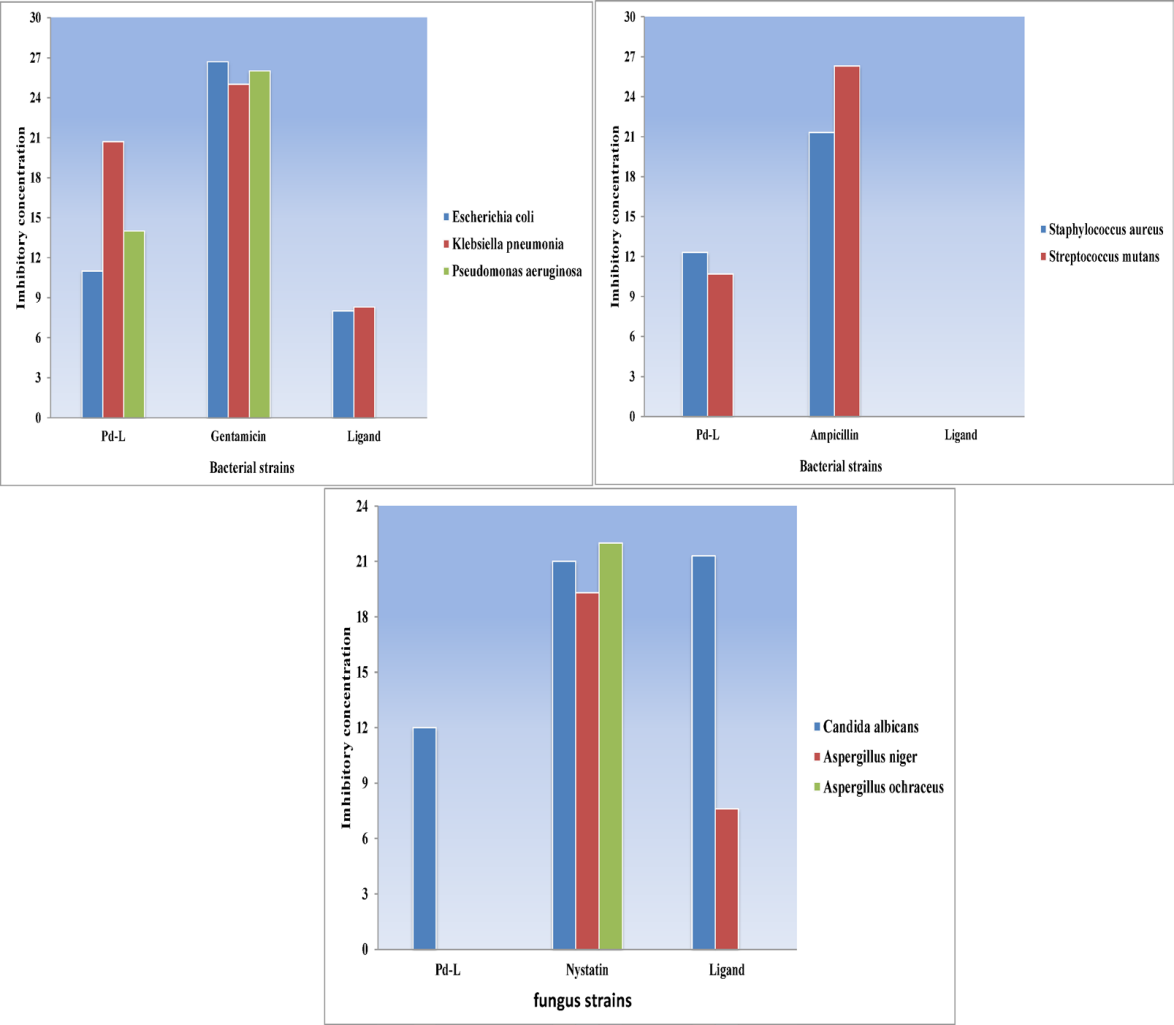
Graphical representation of antibacterial and antifungal activities of the ligand **5** and Pd-complex with gentamicin and nystatin, standard drugs, respectively.

Thiadiazolo[2,3-c][1,2,4] triazine derivative **5** and its palladium (II) complex were subjected to antimicrobial testing by measuring the inhibition zone diameter (IZD) in millimeters^59^ against *Escherichia coli* (ATCC: 10536), *Klebsiella pneumonia* ATCC: 10031), *Pseudomonas aeruginosa* (ATCC: 27853), *Staphylococcus aureus* (ATCC: 13565), *and Streptococcus mutans* (ATCC: 22947) as bacterial species in addition to *Candida albicans* (ATCC: 10231), *Aspergillus niger* (ATCC: 16404), and *Aspergillus ochraceus* (ATCC: 22947) as fungal species. The results were compared with three standard medications: Gentamicin, Ampicillin, and Nystatin, as described in Table 11. The summary of the results, as shown in Figs. 11 and 12, reveals the following findings:

1. Based on the data, it is evident that the free ligand (**5**) exhibits a substantial inhibitory effect against *Escherichia coli*, *Klebsiella pneumoniae*, *Candida albicans*, and *Aspergillus Niger*. However, it remains inactive against Gram (+) bacteria, *Pseudomonas aeruginosa*, and *Aspergillus ochraceus* compared to its corresponding palladium (II) complex. An increased activity of the Pd(II) complex over the ligand can be explained based on Overton’s concept and the Chelation theory^60,61^. On chelation, the polarity of the metal ion will be reduced to a greater extent, attributable to the overlap of the ligand orbital and partial sharing of the positive charge of the ligand orbital and donor groups. The mode of action of compounds may involve forming hydrogen bonds with the active sites of cellular constituents, thereby interfering with normal cell processes.
2. Pd(II)-complex exhibits biological action against the majority of bacterial and fungal species, except *Aspergillus Niger* and *Aspergillus ochraceus* fungi.
3. Neither the ligand nor the Pd (II) complex exhibits any activity against *Aspergillus ochraceus* fungi.
4. The effectiveness of the activity against various microorganisms is contingent upon either the impermeability of microbial cell membranes or disparities in ribosomes within these cells.
5. The solvent control (DMSO) showed no inhibition zone (0 mm) against all tested microorganisms, indicating that the solvent had no antimicrobial activity.

The antimicrobial study was further extended to evaluate the minimum inhibitory concentration (MIC) of the most promising compound against the Gram-negative bacterium Klebsiella pneumoniae. The palladium complex was selected based on its superior activity in the preliminary screening against *K. pneumoniae*. The MIC value was determined to be 250 µg/mL. The standard Gentamicin showed a minimum inhibitory concentration (MIC) value of 62.5 µg/mL.

**Table 11:** Antimicrobial activities are expressed IZD (mm) of the synthesized compounds.

		Tested Samples		Standard drugs	Solvent
		**5**	**Pd complex**	*Gentamicin*	*DMSO*
Gram-negative bacteria	*Escherichia coli*	8.0 ± 1.0	11.0 ± 1.0	26.7 ± 0.6	0.0
	*Klebsiella pneumonia*	8.3 ± 0.6	20.7 ± 0.6	25.0 ± 1.0	0.0
	*Pseudomonas aeruginosa*	No activity	14.0 ± 1.0	26.0 ± 1.0	0.0
Gram-positive bacteria				*Ampicillin*	
	*Staphylococcus aureus*	No activity	12.3 ± 0.6	21.3 ± 0.6	0.0
	*Streptococcus mutans*	No activity	10.0 ± 1.0	26.3 ± 0.6	0.0
Fungi				*Nystatin*	
	*Candida albicans*	8.3 ± 0.6	12.0 ± 1.0	21.0 ± 1.0	0.0
	*Aspergillus niger*	7.7 ± 0.6	No activity	19.3 ± 0.6	0.0
	*Aspergillus ochraceus*	No activity	No activity	22.0 ± 1.0	0.0

### Molecular docking

Molecular docking is a computational technique that simulates the interaction between a small molecule (ligand) and a larger molecule (target), such as a protein or a DNA molecule. It can be used to study how ligands bind to their targets, and to design new drugs or optimize existing ones. One of the most popular molecular docking software programs is MOE. Different algorithms and scoring functions are used to search for the optimal binding pose and the ligand-target complex affinity. Designated compounds are docked using the MOE 2014.010 Package, one of the in silico study tools. Synthesized compounds are targeting ENOYL REDUCTASE [Protein Data Bank (PDB ID: 1LXC)], Fig. 14.

**Fig. 13:**
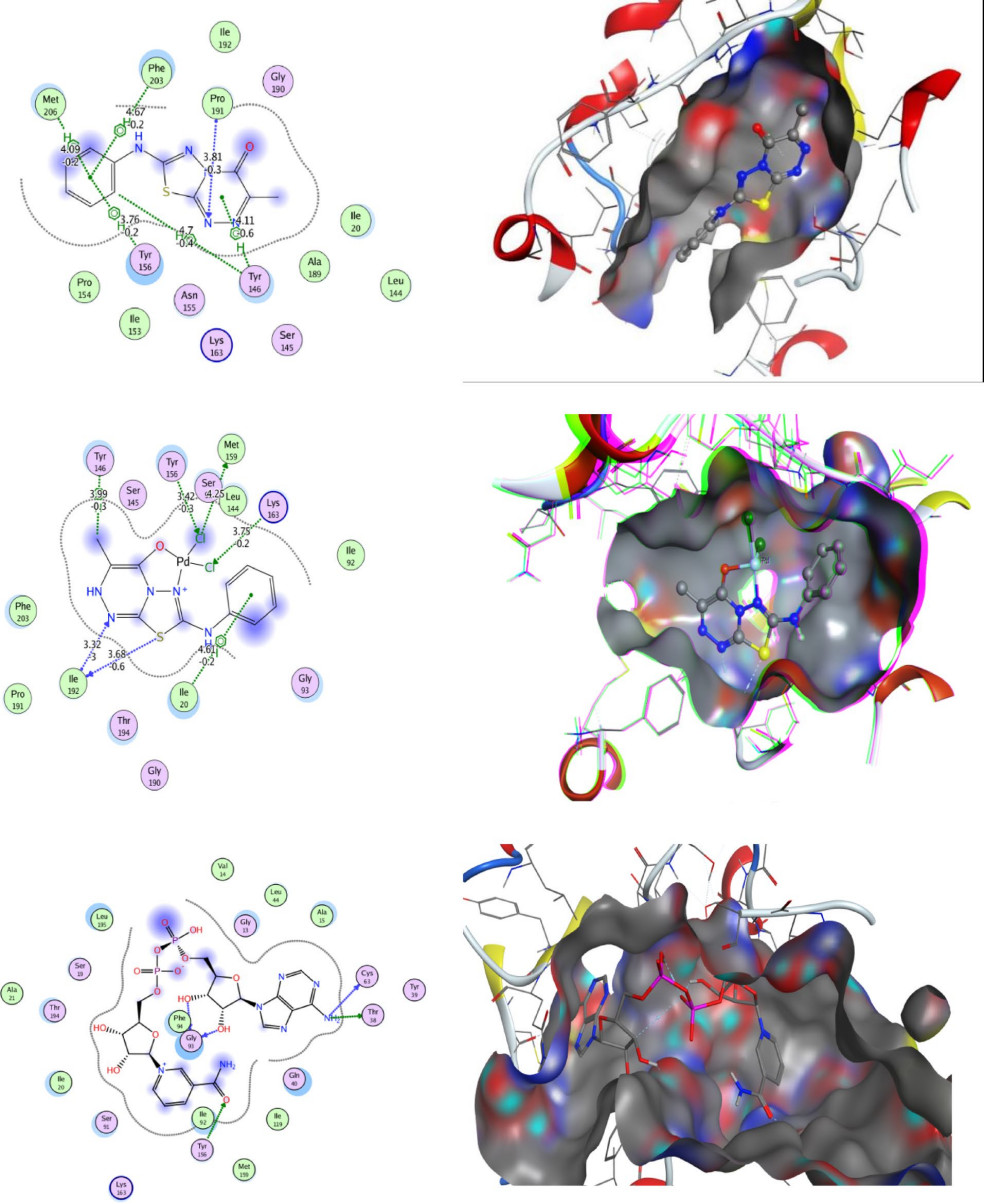

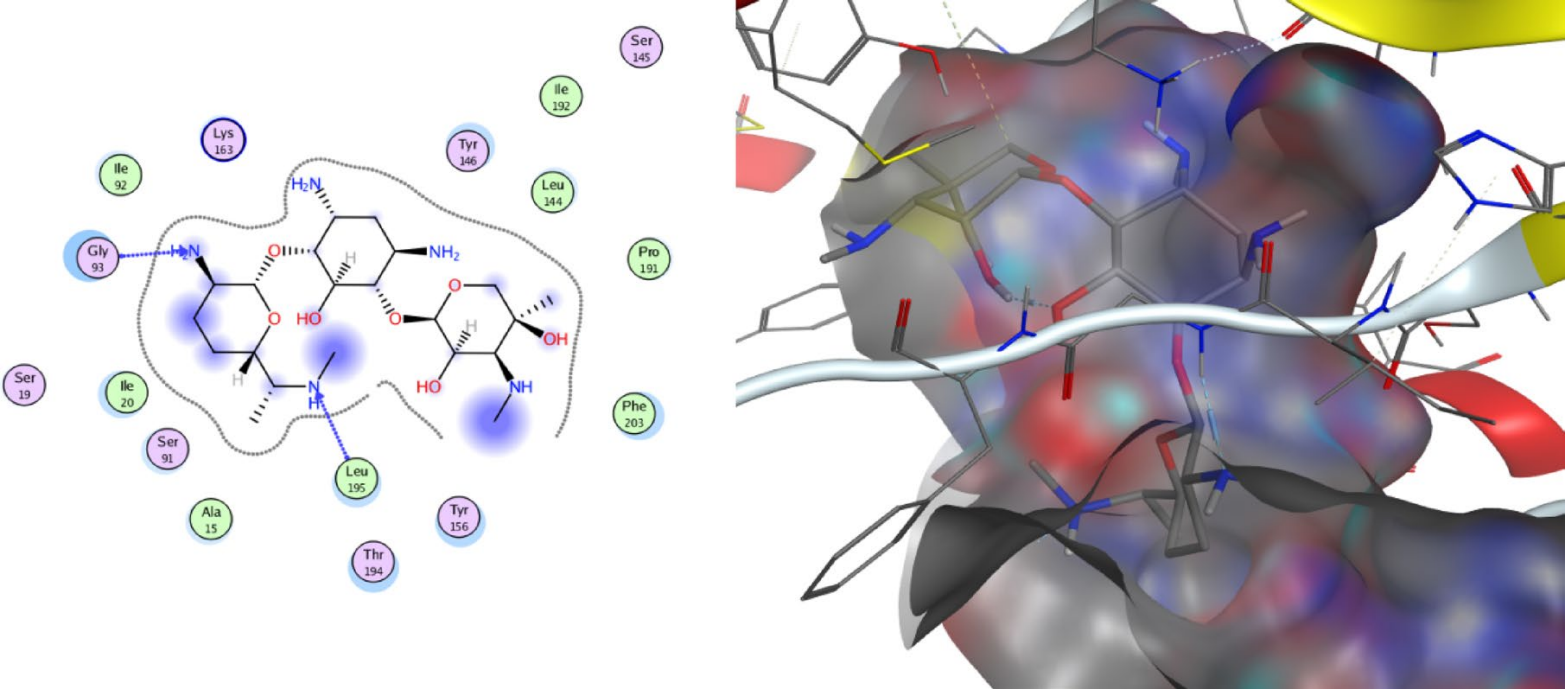
2D images represent the interactions between the compound **5**, Pd(II)-complex, nicotinamide-adenine-dinucleotide and gentamicin with the active binding site residues. A 3D image showing compound **5** and its Pd(II) complex embedded into the active pocket.

The docking results indicated that compound **5** (Table 12; Fig. 13) exhibited a binding energy of − 1.9 kcal/mol (RMSD = 2.1 Å), forming one hydrogen bond and several π–H interactions within the protein binding pocket, confirming its ability to fit within the binding pocket and interact with key residues. Although the binding energy is relatively moderate, it still reflects a spontaneous interaction (ΔG < 0), suggesting possible biological relevance, particularly as an initial scaffold. In comparison, the palladium complex (Table 13; Fig. 13) showed improved binding affinity (− 4.6 kcal/mol, RMSD = 1.1 Å) with multiple hydrogen bonds, indicating enhanced stabilization within the active site. Notably, docking of the reference drug gentamycin under identical conditions demonstrated a stronger binding affinity (− 6.46 kcal/mol, RMSD = 2.04 Å), consistent with its established antibacterial activity. Such differences in binding energies are expected, as docking scores primarily provide relative rather than absolute measures of affinity, and values more negative than − 3 kcal/mol are generally considered indicative of stable interactions, while stronger affinities are associated with values below − 5 kcal/mol^62^. Therefore, the obtained results suggest that the studied compounds, particularly the palladium complex, exhibit promising binding behavior.

‏.

The validation of the docking protocol was performed by redocking the co-crystallized ligand Nicotinamide adenine dinucleotide (NAD) into the active site of Enoyl-[acyl-carrier-protein] reductase. The docking results showed a binding energy of − 8.12 kcal/mol, indicating a favorable interaction within the enzyme active site. The ligand formed key interactions with important amino acid residues, particularly Cys-63 and Thr-38, which help stabilize the ligand within the binding pocket. These results confirm the reliability of the docking protocol and validate its use for further virtual screening of potential inhibitors targeting the enzym.

**Table 12:** Binding energies of palladium (II)-complex.

Ligand	Receptor	Interaction	Distance (Å)	E (kcal/mol)
N 3	CA PRO 191 (A	H-acceptor	3.81	-0.3
C 17	6-ring TYR 146 (A)	H-pi	4.70	-0.4
6-ring	CB TYR 146 (A)	pi-H	4.11	-0.6
6-ring	CD1 TYR 156 (A)	pi-H	3.76	-0.2
6-ring	CE1 PHE 203 (A)	pi-H	4.67	-0.2
6-ring	CE MET 206 (A)	pi-H	4.09	-0.2

**Table 13:** Binding energies of palladium (II)-complex.

Ligand	Receptor	Interaction	Distance (Å)	E (kcal/mol)
S 7	O ILE 192 (A)	H-donor	3.68	-0.6
CL 13	SD MET 159 (A)	H-donor	4.25	0.0
N 3	N ILE 192 (A)	H-acceptor	3.32	-3.0
CL 13	OH TYR 156 (A)	H-acceptor	3.42	-0.3
CL 14	CE LYS 163 (A)	H-acceptor	3.75	-0.2
C 21	6-ring TYR 146 (A)	H-pi	3.99	-0.3
6-ring	CD1 ILE 20 (A)	pi-H	4.61	-0.2

### Anticancer activity profile with docking study

**Fig. 14:**
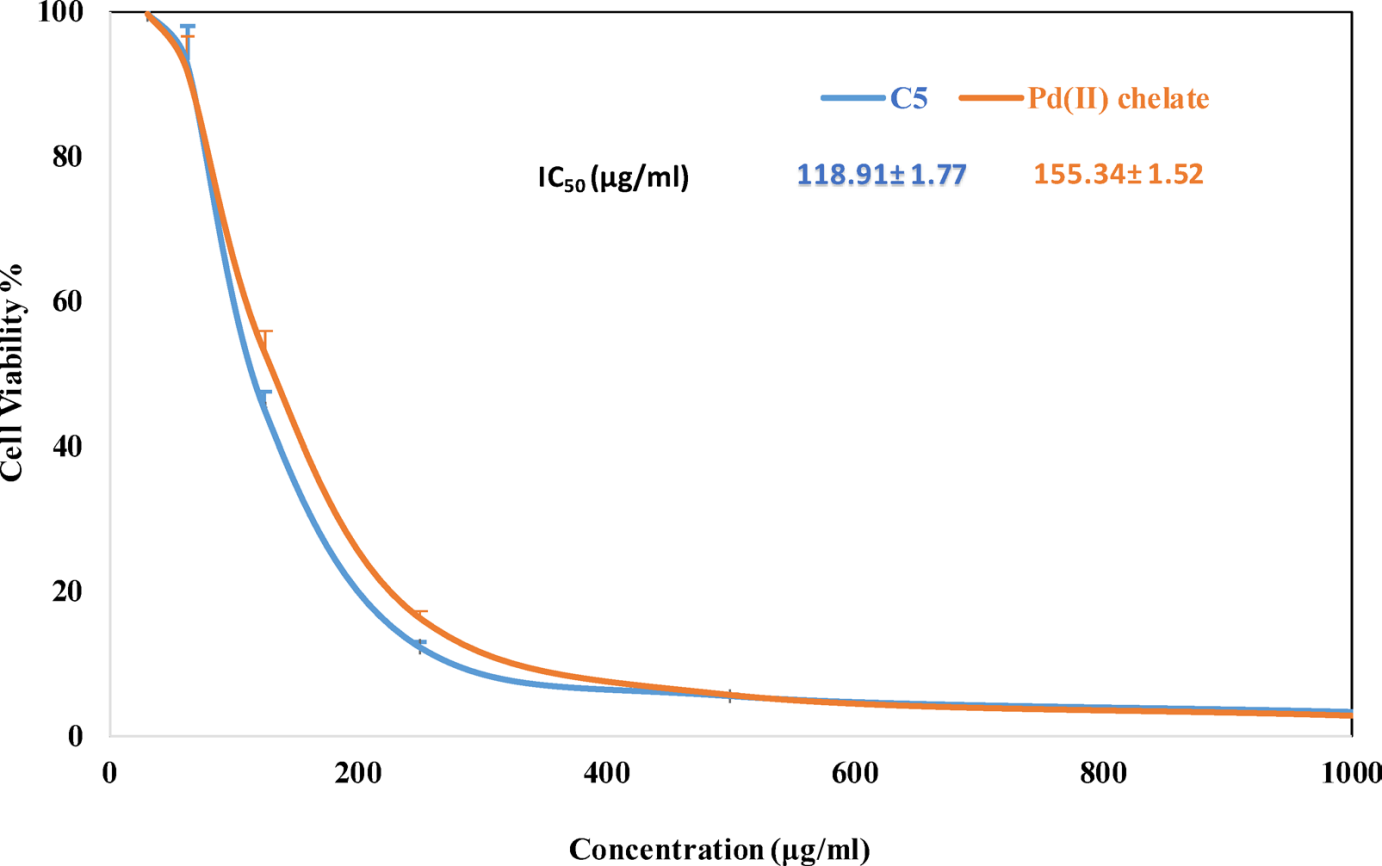
Surviving fraction of ligand **5** and its Pd(II)-complex at different concentrations (µg/mL) on HepG2 human liver cancer cells with IC_50_ values.

**Fig. 15:**
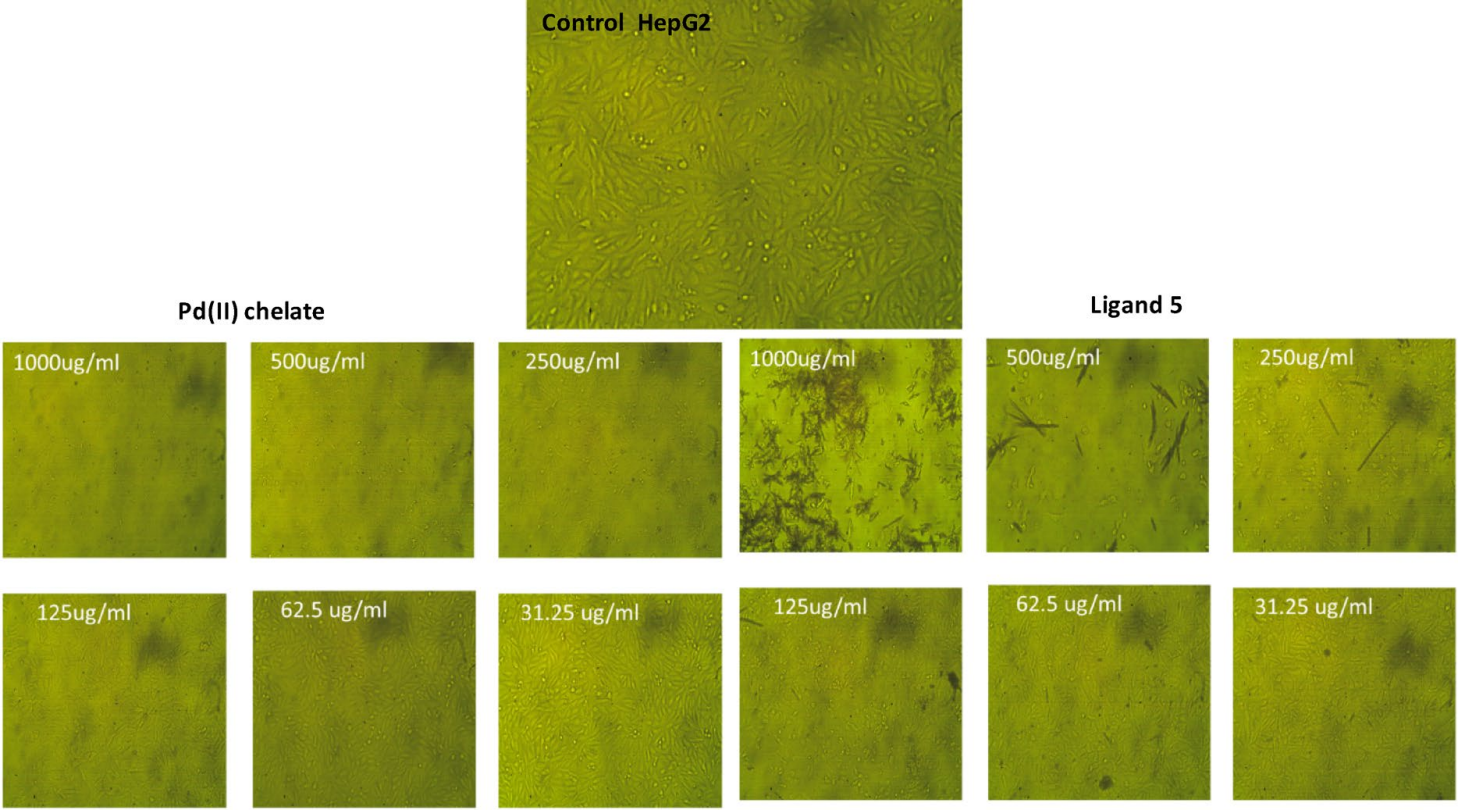
Cytotoxic effect of investigated compounds against HepG2 at different concentrations.

The surviving fractions of ligand **5** and its Pd(II) complex at different concentrations along with their IC_50_ values against HepG2 are shown in Fig. 14. By definition, IC_50_ is the concentration of the drug at which 50% of the target is inhibited. Therefore, the lower the IC_50_ of the drug, the less of this drug is needed to achieve the desired effect. Although it is known that the Pd compounds reveal more anticancer activity than their parent ligand, the triazine ligand exhibits more activity against liver cancer than the Pd complex. Since Pd(II) is isoelectronic to platinum (II) and tetra-coordinate, where the Pd(II) complex is in the same square-planar geometry as cisplatin, the palladium (II) compounds act as potential anticancer agents. It may be due to the difficulty of the aquation mechanism of cisplatin and its similar compounds, which involve the replacement of the two chloride ions by the water molecules within cancer cells. The cytotoxic effects of the investigated compounds against HepG2 at different concentrations is presented in Fig. 15. The cytotoxicity was evaluated against HepG2 cells using the MTT assay. The reference drug doxorubicin exhibited an IC_50_ value of approximately 7.5 ± 0.5 µg/mL.

**Fig. 16:**
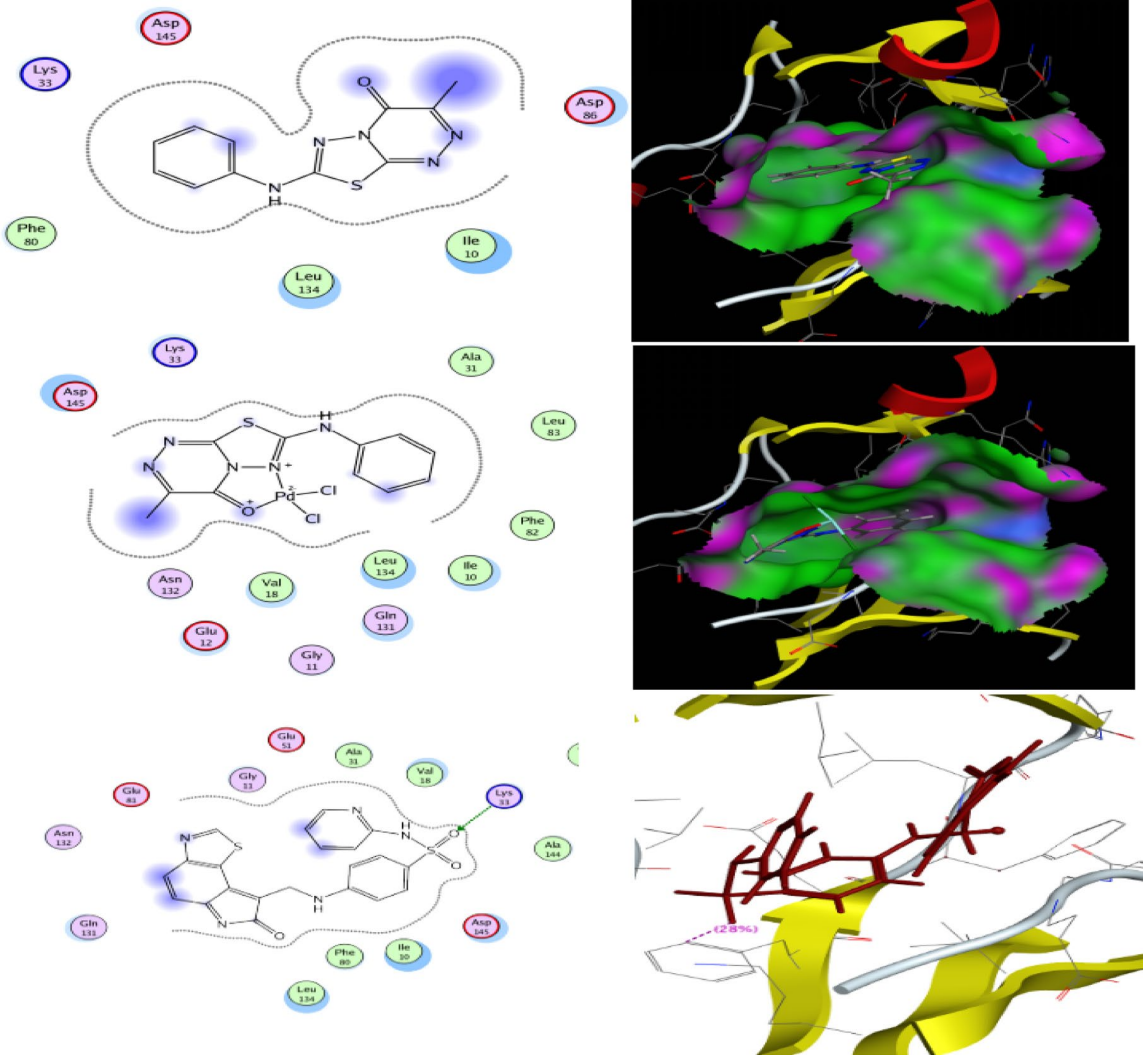
EGFR tyrosine kinase receptor docking results with all synthesized compounds.

In this investigation, docking tests are being conducted to determine the type and degree of interactions between the synthesized proposed compounds and the Cyclin-dependent kinases (CDK-2) enzyme. The CDKs are potential targets for anticancer medications because they interfere with CDK activity, leading to cell death and upsets cell cycle regulation in cancer cells. The **L** and **Pd-**complex interact with the CDK2 active site, with scoring energies and RMSD of -3.77 Kcal/mol (2.72) and − 6.58 Kcal/mol (1.00), respectively, as shown in Fig. 16. Both compounds exhibit comparable activity against the selected protein. The expected docking interaction corroborated the experimental data. The co-crystallized ligand in the CDK-2 enzyme receptor (PDB ID: 1FVV) was 4-[(7-OXO-7 H-THIAZOLO[5,4-E]INDOL-8-YLMETHYL)-AMINO]-N-PYRIDIN-2-YL-BENZENESULFONAMIDE. The validation was performed by docking the ligand in its binding pocket. The validation revealed a good pose with a high docking score and an RMSD of less than 2 Å for the side-chain donor interaction type. The hydrogen bond was formed through the oxygen sulfonamide group of the co-crystallized ligand with the NH_2_ of Lys 33 amino acid, as seen in Fig. 16.

### Effect of compounds on the denaturation inhibition of protein

To further evaluate the pharmacological potential of the synthesized compounds, their ability to inhibit protein denaturation was investigated. Both the ligand **5** and its Pd(II) complex exhibited concentration-dependent inhibition of protein denaturation. The ligand **5** showed inhibition percentages of 0%, 27.5%, 45.0%, 62.8%, and 74.0% at concentrations of 0, 5, 10, 20, and 30 µg mL^-1^, respectively. In comparison, the Pd(II) complex exhibited higher inhibition values of 0%, 32.0%, 50.0%, 68.1%, and 83.2% at the same concentrations. The IC_50_ values were estimated to be approximately 11.1 µg mL^–1^ for the ligand **5** and 10.0 µg mL^−1^ for its Pd(II) complex, indicating a higher inhibitory potency of the metal complex. The enhanced inhibitory effect of the Pd(II) complex suggests that metal coordination improves the ability of the ligand to stabilize protein structure against denaturation, which may be attributed to stronger interactions between the metal complex and the protein. Knowing that the protein denaturation activity indicated that the standard drug ibuprofen achieved an inhibition percentage of 66.66%.

## Conclusions

The ligand; 3-methyl-7-(phenylamino)-4 H-[1,3,4] thiadiazolo[2,3-c][1,2,4] triazin-4-one was successfully synthesized using an improved synthetic method. This was followed by the successful preparation of a Pd(II)-complex, yielding a dichloro complex structurally analogous to the cisplatin drug scaffold. The synthesized compounds were thoroughly characterized using various analytical techniques. Density Functional Theory (DFT) calculations supported the formation of the ligand by confirming its stability and favoring the targeted structure. DFT also confirmed the square planar geometry of the Pd(II)-complex. Both the ligand and its Pd(II)-complex demonstrated notable antimicrobial activity, particularly against *Klebsiella pneumoniae*, indicating their potential as alternative antibacterial agents. Moreover, both compounds exhibited synergistic effects with significant anticancer and anti-inflammatory activities. Molecular docking studies further explored their interactions with selected target proteins, including EGFR tyrosine kinase and GlcN-6-P synthase, highlighting their potential biological relevance.

## Supplementary Information

Below is the link to the electronic supplementary material.

Supplementary Material 1

## Data Availability

All data generated or analysed during this study are included in this published article [and its supplementary information files].
