# Supplementary material for: Synthesis, biological activities, DFT, and molecular docking of 1,3,4-thiadiazolo[2,3-c]-1,2,4-triazine-Palladium (II) complex
Source: Sci Rep. 2026 Jun 4;16:17336. doi: 10.1038/s41598-026-53122-1 (PMC13237342; doi:10.1038/s41598-026-53122-1)
Supplement: Supplementary file 1 — Supplementary Material 1 [file 41598_2026_53122_MOESM1_ESM.docx]

# **1. Experimental**

**1.1. Chemistry Section**

**1.1.1. Instruments.**

Melting points were measured on a Gallenkamp melting point apparatus. TLC was performed on a silica gel of aluminium 60 F254 (Merck) sheet, and detected by short UV light. The infrared spectra were recorded on KBr disks on a Pye Unicam SP 3300 and Shimadzu FT-IR 8101 PC infrared spectrophotometers. The NMR spectra were measured on a Varian Mercury VX-300 NMR spectrometer. ^1^H spectra were run at 300 MHz, and ^13^C spectra were run at 75.46 MHz in dimethyl sulfoxide (DMSO-*d_6_*). Chemical shifts were related to those of the solvent. Mass spectra were recorded on a Shimadzu GCMS-QP 1000 EX mass spectrometer at 70 eV. Elemental analyses and antimicrobial evaluations were carried out at the Microanalytical Center of Cairo University, Egypt. X-ray diffraction (XRD) analysis was carried out with a Bruker LynxEye detector utilizing Cu-Kα radiation (wavelength = 1.5406 Å). To correct the instrumental broadening, the measured peak widths (FWHM) were corrected by subtracting the instrument’s contribution before applying the Scherrer equation. This is done by measuring a well‑crystallized standard material under the same XRD conditions to obtain the instrumental broadening (β_instr). The true sample broadening (β_sample) is then calculated as β_sample = √(β_obs² − β_instr²), where β_obs is the observed FWHM. The corrected broadening β_sample is used in t = 0.9 λ/(β_sample cos θ) to determine the crystallite size, ensuring the contribution of the instrument is removed

**1.2. Biological evaluation**

**1.2.1. Antibacterial Study**

***Reagents*:** Dimethylsulphoxide (DMSO), Gentamicin (C_21_H_43_N_5_O_7_, 477.596 g/mol), Ampicillin (C_16_H_19_N_3_O_4_S, 349.406 g/mol), and Nystatin (C_47_H_75_NO_17_, 926.107 g/mol) antibiotics were obtained from Sigma Chemical Co. (St. Louis, MO, USA).

***Bacterial cultures*:** The different strains are *Escherichia coli* (ATCC: 10536), *Klebsiella pneumonia* (ATCC: 10031), *Pseudomonas aeruginosa* (ATCC: 27853), *Staphylococcus aureus* (ATCC: 13565), and *Streptococcus mutans* (ATCC: 22947)) as bacterial species in addition to *Candida albicans* (ATCC: 10231), *Aspergillus Niger* (ATCC: 16404), and Aspergillus ochraceus (ATCC: 22947) as a fungal species.

***Antimicrobial susceptibility*:** Antimicrobial susceptibility of the pathogenic selective strains was assessed by the agar well diffusion method [32] against the target compounds, paralleled with Gentamicin, Ampicillin, and Nystatin as standard medications. The diameter of the zones of inhibition (ZOI, mm) was measured accurately to indicate antibacterial and antifungal activity.

***In vitro antibacterial and antifungal assay:*** The sterilized media was poured onto the sterilized Petri dishes (20~25 mL), each Petri dish) and allowed to solidify at room temperature. The microbial suspension was prepared in sterile saline equivalent to a McFarland 0.5 standard solution (1.5 x 10^5^ CFU mL^-1^), and its turbidity was adjusted to OD = 0.13 using a spectrophotometer at 625 nm. Optimally, within 15 minutes of adjusting the turbidity of the inoculum suspension, a sterile cotton swab was dipped into the adjusted suspension, then flooded onto the dried agar surface, and allowed to dry for 15 minutes with the lid in place. Wells of 6 mm diameter were made in the solidified media with the help of a sterile borer. A solution of 100 μL of the tested compound was added to each well using a micropipette. The plates were incubated at 37°C for 24 hrs.

**1.2.2. Cell viability assessment assay**

In this investigation, the cell lines (HepG2) and HFF-1 used in this study were commercially obtained from VACSERA (Holding Company for Biological Products & Vaccines, Egypt). HepG2 was cultured in a cell incubator under conditions of 5% CO_2_ at 37 °C, using DMEM (90%) supplemented with FBS (10%) and penicillin/streptomycin (1%). HFF-1 cells were detached using trypsin, and suspended in cold PBS, and viable cells were identified by staining with 0.1% trypan blue and microscopic counting before experimentation. To evaluate cell viability, the MTT method was employed [33]. Upon reaching a confluence of approximately 80–90%, cancer cells were seeded into a 96-well plate at a density of 1× 10^5^ cells/well and allowed to adhere for 24 hours. Following this incubation period, the cells were rinsed with PBS, treated with the tested compounds containing DMEM medium at varying concentrations, and further incubated for an additional 24 hours. Subsequently, the MTT assay was applied, incubating the cells for 4 hours, followed by the addition of 100 μl of DMSO after 5 hours. The plate was then assessed using an ELISA microplate reader at 570 nm (ELx808i, BioTek Instruments, USA), and the percentage of viable cells was calculated using the formula:

$$\% Growth inhibition rate = 100- \frac{Total number of viable cells per milliliter of aliquot}{Total number of cells per milliliter of aliquot}x100$$

**1.2.3. Bovine serum albumin denaturation inhibition measurement**

Screening for protein denaturation inhibitors is crucial in anti-inflammatory screening investigations. A protein denaturation test was performed as described by Gambhire et al [34] with slight modification. In a reaction volume of 5 ml, composed of 200 µl of 1.0 % of BSA (Bovine Serum Albumin) and PBS buffer (4.78 ml, pH 7.4), a sample concentration of 10 µg/ml was utilized. After the reaction solution was incubated at 37°C for 15 min, the reaction was heated at 70°C for 5 min, the tube was allowed to cool, and the developed turbidity was measured spectrophotometrically at 660 nm. The control consisted of phosphate buffer solution and BSA without the tested sample. The % inhibition of denaturation was calculated based on the following equation:

% inhibition of denaturation = $\frac{1-Absorbance of the tested sample}{Absorbance of the control}\times100$

**Statistical analysis**

Differences between samples within the same type of bacteria (or fungi) were analyzed using one-way analysis of variance (ANOVA), followed by Duncan's multiple comparisons tests using SPSS package version “22” for Windows. Values are represented as mean ± S.E., and p<0.05 was considered statistically significant, p<0.01 was considered highly significant, and p<0.001 was considered very highly significant.


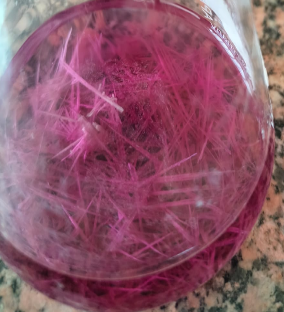


**Figure S1. The separated crystals of** **4-amino-6-methyl-3-thioxo-3,4-dihydro-1,2,4-triazin-5(2H)-one (1).**


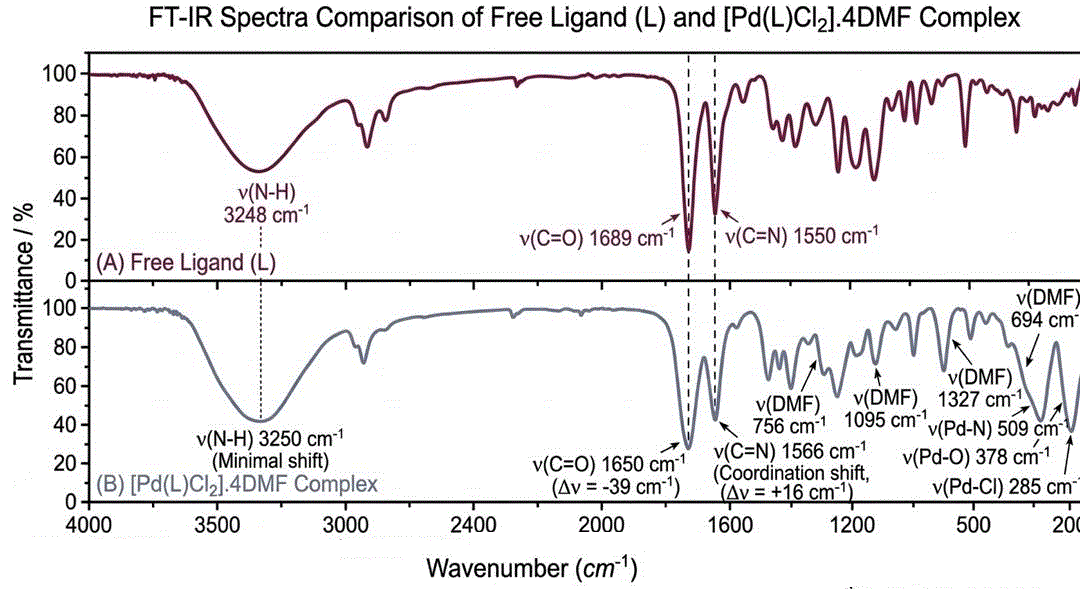


**Figure S2. FTIR spectra of ligand (5) and its Pd(II) complex.**

**Scheme S1 the suggested fragments of Pd(II) complex.**

**Table S1: Mass spectral fragmentations of Pd-L chelate**

| **Compound** | **Fragmented ion** | **m/e^+^ values** | | **Lost Spices** |
| --- | --- | --- | --- | --- |
|  |  | **Calc.** | **found** |  |
| **Pd-L complex** | [Pd(C_11_H_9_N_5_OPdS)Cl_2_]​⋅4DMF  [Pd(C_10_​H_6_​N_5_​OS)Cl_2_​⋅3DMF]^+^  [Pd(C_10_​H_6_​N_5_​OS)⋅3DMF]^+^  [Pd(C_7_​H_6_​N_5_​OS)⋅2DMF]^+^  [Pd(C_4_​H_2_​N_5_​OS)⋅ DMF. C_2_H_7_N ]^+^  [Pd(C_4_​H​N_4_​S)⋅ C_2_H_7_N ]^+^  [Pd⋅ C_2_H_4_N]^+^ | 728.99  639.85  570  460.45  392.22  288.37  148.74 | 728.42  640.86  571.54  460.22  392.46  288.80  148.94 | **-**  DMF + CH_3_  Cl_2_  DMF + C_3_H_2_  C_4_H_4_O  DMF + NH + O  C_4_H_4_N_4_S |
